# Nanotools for Sepsis Diagnosis and Treatment

**DOI:** 10.1002/adhm.202001378

**Published:** 2020-11-25

**Authors:** Lana Papafilippou, Andrew Claxton, Paul Dark, Kostas Kostarelos, Marilena Hadjidemetriou

**Affiliations:** ^1^ Nanomedicine Lab Faculty of Biology Medicine and Health AV Hill Building The University of Manchester Manchester M13 9PT UK; ^2^ Department of Critical Care Salford Royal Foundation Trust Stott Lane Salford M6 8HD UK; ^3^ Manchester NIHR Biomedical Research Centre Division of Infection Immunity and Respiratory Medicine University of Manchester Manchester M13 9PT UK; ^4^ Catalan Institute of Nanoscience and Nanotechnology (ICN2) Campus UAB Bellaterra Barcelona 08193 Spain

**Keywords:** diagnosis, nanotechnology, nanotheranostics, sepsis, treatment

## Abstract

Sepsis is one of the leading causes of death worldwide with high mortality rates and a pathological complexity hindering early and accurate diagnosis. Today, laboratory culture tests are the epitome of pathogen recognition in sepsis. However, their consistency remains an issue of controversy with false negative results often observed. Clinically used blood markers, C reactive protein (CRP) and procalcitonin (PCT) are indicators of an acute‐phase response and thus lack specificity, offering limited diagnostic efficacy. In addition to poor diagnosis, inefficient drug delivery and the increasing prevalence of antibiotic‐resistant microorganisms constitute significant barriers in antibiotic stewardship and impede effective therapy. These challenges have prompted the exploration for alternative strategies that pursue accurate diagnosis and effective treatment. Nanomaterials are examined for both diagnostic and therapeutic purposes in sepsis. The nanoparticle (NP)‐enabled capture of sepsis causative agents and/or sepsis biomarkers in biofluids can revolutionize sepsis diagnosis. From the therapeutic point of view, currently existing nanoscale drug delivery systems have proven to be excellent allies in targeted therapy, while many other nanotherapeutic applications are envisioned. Herein, the most relevant applications of nanomedicine for the diagnosis, prognosis, and treatment of sepsis is reviewed, providing a critical assessment of their potentiality for clinical translation.

## Graphical abstract

Sepsis remains among the leading causes of morbidity and mortality worldwide. The lack of rapid, sensitive, and specific tests for sepsis impedes early diagnosis and results in delayed and ineffective antimicrobial treatments. This review covers the development of nanoparticle‐based technologies for the diagnosis, prognosis, and treatment of sepsis, providing a critical assessment of their clinical translatability.

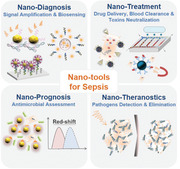

## 1 Introduction

Sepsis is defined as “life‐threatening organ dysfunction caused by a dysregulated host response to an infection.”^[^ ^1^ ^]^ Today, sepsis is among the leading causes of morbidity and mortality worldwide in intensive care units (ICU)^[^ ^1^, ^2^ ^]^ with its survival rate for severe forms decreasing by as much as 8% every hour^[^ ^3^ ^]^ before the appropriate antibiotic therapy is initiated.^[^ ^4^ ^]^

Diagnosing sepsis as early as possible is critically important as delays in administering appropriate treatment can precipitously affect outcome. Currently, diagnosis relies on clinical manifestations and blood tests for the detection of inflammation response‐related blood biomarkers, such as CRP and PCT. These clinically available protein biomarkers however, lack specificity^[^ ^5^ ^]^ making sepsis recognition in its early stages extremely difficult. Microbiological culture techniques remain the current gold standard method to identify causative pathogen phenotypes. Nonetheless, they can take up to 72 h and are often associated with a high false negative rate.

Sepsis is a medical emergency in which time is a crucial factor. Delays in treatment can lead to multiple organ failure and death. As such, due to the high mortality rate associated with delayed treatment and the lack of specific diagnostic and therapeutic guidance, clinicians empirically administer broad‐spectrum antibiotics as early as possible.^[^ ^6^ ^]^ The use of broad‐spectrum agents however, may not be as efficacious as therapeutics targeted against specific pathogen phenotypes.^[^ ^7^ ^]^ Another challenge in the clinical setting is the diagnostic uncertainty in differentiating septic patients from those suffering from noninfectious systemic inflammation. The clinical signs and symptoms of sepsis in its early stages mirror those of noninfectious inflammation and this leads to antibiotics being administered to patients with sterile inflammation or viral infections. The overuse of antibiotics is often associated with unwanted side effects, such as the proliferation of antimicrobial resistant organisms and patient toxicity. Moreover, as sepsis treatment is primarily restricted to antibiotics, clinicians rely on their therapeutic efficacy. Nevertheless, the acute alterations in physiology during sepsis can result in poor pharmacokinetics and unsuccessful drug delivery.^[^ ^8^ ^]^

Considering all the pitfalls associated with sepsis, there is an urgent need to develop rapid, sensitive and pathogen‐specific detection tests, as well as new antimicrobial strategies. Several promising targets have been proposed as potential means of sepsis detection and therapy, but they have been unable to step from research level to clinical implementation, due to difficulties in modeling the highly variable septic responses in preclinical systems.^[^ ^9^ ^]^ Sepsis involves the activation of a combination of different pathological pathways and therefore there are no adequately representative animal models that can reflect sepsis heterogeneity and sufficiently simulate its complexity.^[^ ^9^ ^]^ To date, there is a limited portfolio of preclinical data showing enhanced sensitivities and specificities when compared to clinically used technologies, and this poses significant challenges in clinical trials.^[^ ^10^ ^]^

**Figure 1:**
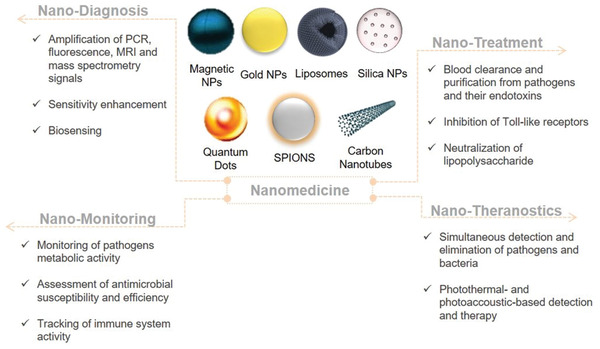
Nano‐toolbox for sepsis diagnosis, monitoring, and treatment. Type of nanoparticles used for the management of sepsis and the advantages coming from their exploitation for disease diagnostic, monitoring, and therapeutic and theranostic purposes.

The emergence of nanotechnology and its incorporation within medicine have revolutionized the traditional pharmaceutical and medical world.^[^ ^11^ ^]^ The field has already proposed innovative technological solutions to improve current diagnostic and therapeutic management of several pathologies.^[^ ^12^, ^13^, ^14^, ^15^, ^16^ ^]^ Strikingly, even though nanotechnology counts only several decades, more than 200 nanomedicine constructs are under clinical investigation or clinical use.^[^ ^17^ ^]^ The employment of nanoparticles (NPs) for diagnostic and therapeutic purposes offers great potential, owing to their tunable properties (e.g., size, charge, surface chemistry, shape, and composition) and their capacity for surface functionalization (with ligands, antibodies, and targeting molecules), which allows targeted and selective binding. Additionally, nanoscale drug delivery systems can be engineered to improve the biodistribution of already existing therapeutics by improving the efficacy, stability and bioavailability of the drug at the target site.^[^ ^18^ ^]^ All these together, have prompted the research for “nano” strategies (**Figure** **1**) that could help clinicians in addressing the main roadblocks associated with sepsis.

This review will highlight the current state‐of‐the‐art on novel nanotechnology‐enabled approaches for the diagnosis, treatment, and monitoring of sepsis and will discuss the future development of advanced and clinically applicable nanotheranostic platforms.

## 2 Nanodiagnostic Technologies for Sepsis

Rapid, sensitive and specific detection of the infectious pathogen is crucial for the clinical progression and outcome of a septic patient. Current molecular techniques employed for microbial infection diagnosis, including enzyme‐linked immunosorbent assay (ELISA) and polymerase chain reaction (PCR) are thought to offer high sensitivity and reproducibility. However, they require experienced personnel, pose a high risk of sample contamination and lack versatility needed in medical diagnosis.^[^ ^19^ ^]^

Nanotechnology can aid in the development of fast, sensitive, and accurate methods for sepsis detection.^[^ ^20^, ^21^ ^]^ Several NPs have been investigated to allow the diagnosis of sepsis‐related microbial infections, such as magnetic (MNPs), gold (AuNPs), fluorescent (silica and quantum dots QDs), and lipid‐based NPs.^[^ ^22^, ^23^, ^24^, ^25^, ^26^ ^]^ Most of them are primarily used as contrast agents and biosensors to facilitate the detection of either proteins and nucleic acids associated with sepsis (CRP, PCT, and miRNA), pathogenic DNA or bacterial cells by amplifying signals. The main techniques studied for NP‐enabled sepsis diagnosis are based on PCR, colorimetric biosensing, surface‐enhanced Raman scattering (SERS), lens‐free interferometric microscopy (LIM), mass spectrometry (MS), and magnetic resonance imaging (MRI) (**Table** **1**, **2** and Figure 1). Herein, we strictly focus on the “nano”diagnosis of sepsis and therefore of microbial infections induced by certain pathogens (e.g., bacteria and fungi) which are frequently encountered in sepsis, such as *Staphylococcus aureus, Klebsiella pneumoniae*, and *Escherichia coli*, to name but a few.

**Table 1:** Nanotechnology‐based approaches for sepsis diagnosis and monitoring

Technique	Used nanoparticle	Aim and role	Ref.
Colorimetric biosensing (surface‐enhanced plasmon resonance effect)	AuNPs	Naked eye detection of pathogens and metabolic activity assessment of pathogens	^[^ ^29^, ^30^, ^31^, ^32^, ^35^, ^36^, ^37^, ^38^, ^72^ ^]^
Lens‐free interferometric microscopy (LIM)	Au nanohole substrates	Enhancement of optical signals	^[^ ^39^, ^40^ ^]^
Fluorescence resonance energy transfer (FRET)	Silica NPs, QDs	Fluorescent signal amplification	^[^ ^48^, ^49^, ^50^, ^51^, ^52^ ^]^
Magnetic resonance imaging (MRI)	Magnetic NPs, SPIONs	Contrast agents	^[^ ^55^, ^56^, ^57^, ^58^, ^59^, ^61^, ^64^, ^74^ ^]^
Surface‐enhanced Raman scattering (SERS)	Au‐coated MNPs, Magnetic core–polymeric shell biomimetic NPs	Sepsis biomarkers capturing	^[^ ^62^, ^63^ ^]^
Mass spectrometry MS)	MNPs, Liposomes	Mass spectrum enrichment	^[^ ^65^, ^70^ ^]^
Polymerase chain reaction (PCR)	SPIONs, AuNPs, MNPs	DNA amplification	^[^ ^30^, ^31^, ^32^, ^34^, ^35^, ^36^, ^55^, ^56^, ^57^, ^59^, ^60^ ^]^

**Table 2:** Comparison of nanodiagnostic technologies for sepsis

Used nanoparticle	Size [nm]	Target molecules	Sample	Process time	Technique	Sensitivity/Limit of Detection (LOD)	Ref.
Mercaptoalkyloligonucleotide‐conjugated AuNPs	13	DNA	Salmon sperm DNA	–	Colorimetric‐based PCR	10 fmol	^[^ ^31^ ^]^
DNA‐modified AuNPs	50	*mecA* gene of *S. aureus*	Genomic DNA from cultured bacterial cells	2 h	Colorimetric‐based PCR	333 zmol or 2 × 10^5^ molecules	^[^ ^32^ ^]^
Oligonucleotide‐functionalized AuNPs and MMPs	13	Proteins and DNA	Buffers, human cerebral spinal fluid and serum	9–10 h	PCR	Proteins: 10^−18^ m DNA: 10^−19^ m	^[^ ^34^ ^]^
Silver‐enhanced AuNPs Verigene	13–20	Gram‐positive bacteria and DNA	Blood culture	2.5 h	PCR	≈10^5^ CFU mL^−1^	^[^ ^35^ ^]^
Nucleotide‐labeled AuNPs and vancomycin‐conjugated magnetic beads	20	*E. coli, K. pneumonia, P. aeruginosa*, and *S. aureus*	Cultured bacterial cells	25 min	Colorimetric‐based integrated microfluidic device	10^2^ CFU mL^−1^	^[^ ^36^ ^]^
AuNPs	100	*P. mirabilis*	Human urine	40 min	Colorimetric	10 CFU mL^−1^	^[^ ^37^ ^]^
AuNPs	45–50	IL‐6	IL‐6 buffer solution and IL‐6 spiked blood	17 min	Colorimetric‐based mobile biosensor	Buffer: 0.1 pg mL^−1^ Blood: 12.5 pg mL^−1^	^[^ ^38^ ^]^
Au nanohole substrate	200	*E. coli*	Human plasma	40 min	Interferometric microscopy	400 CFU mL^−1^	^[^ ^39^ ^]^
Au nanohole substrate	200	CRP, IL‐6, and miRNA‐16	Protein‐ and miRNA‐spiked PBS	–	Interferometric microscopy	CRP: 18 µg mL^−1^ IL‐6: 88 µg mL^−1^ miRNA‐16: 6 µg mL^−1^	^[^ ^40^ ^]^
Oligonucleotide‐AuNP‐conjugated PS nanobeads	200	*S. aureus*	DNA	100 min	Diffusometric sensing	10 × 10^−12^ m	^[^ ^41^ ^]^
Mannose carbon QDs	3	*E. coli* K12 strain	Cell culture and human urine	1 h	Fluorescence	Cell culture: 450 CFU mL^−1^ Human urine: 10^3^ CFU mL^−1^ in	^[^ ^48^ ^]^
CdSe‐QDs	8	CRP and IL‐6	CRP and IL‐6 spiked PBS (10% serum)	30 min	Fluorescence‐based LFA	CRP: 42.5 × 10^−9^ m IL‐6: 0.21 × 10^−12^ m	^[^ ^49^ ^]^
Silica NPs	60	*E. coli* O157:H7 cells	Cultured bacterial cells	20 min	Fluorescence	1 bacterium/100 µL sample	^[^ ^50^ ^]^
Silica NPs	60	*E. coli, S. aureus*, and *S. typhimurium*	Cultured bacterial cells	30 min	Fluorescence	–	^[^ ^51^ ^]^
Silica MNPs and fluorophore‐loaded silica NPs	187	*MNase of S. aureus*	Whole blood	10 min	Fluorescence	682 cells mL^−1^	^[^ ^52^ ^]^
SPIONs	800	5 *Candida* species	Whole blood	3–5 h	PCR	1–3 CFU mL^−1^	^[^ ^55^, ^56^ ^]^
MNPs	<10	*S. aureus*	Cultured bacteria spiked media	1 h	Microfluidic chip‐based μHall device	≈10 cells/1 µL	^[^ ^58^ ^]^
MNPs	20	16S rRNA of 13 bacterial species	Cells and whole blood	2 h	PCR and micronuclear MRI	DNA: 0.5 × 10^−12^ m or 1–2 bacteria/10 mL of blood	^[^ ^59^, ^61^ ^]^
Au‐coated MNPs	20	CRP, PCT, and sTREM‐1 proteins	Human serum	–	SERS‐based immunoassay	CRP: 27 × 10^−12^ m PCT: 103 × 10^−12^ m sTREM‐1: 78 × 10^−12^ m	^[^ ^62^ ^]^
Magnetic core–polymeric shell biomimetic NPs	1000	*S. aureus*	Bacterial cell culture	–	SERS	10 CFU mL^−1^	^[^ ^63^ ^]^
HA‐coated DTPA‐Gd SPIONs	12	ROS	LPS‐induced sepsis mice	20 min	MRI	0.2 × 10^−6^ m	^[^ ^64^ ^]^
(3‐Aminopropyl)triethoxysilane modified MNPs	<15	PBPs from *S. aureus* and *E. coli*	Bacterial cell culture	–	MALDI‐MS	10^3^–10^4^ CFU mL^−1^	^[^ ^65^ ^]^
AmBisome Liposomes	100	Unknown protein biomarkers	Human plasma	–	LC‐MS/MS	–	^[^ ^70^ ^]^

### 2.1 Gold Nanoparticle (AuNP)‐Enabled Sepsis Diagnosis

**Figure 2:**
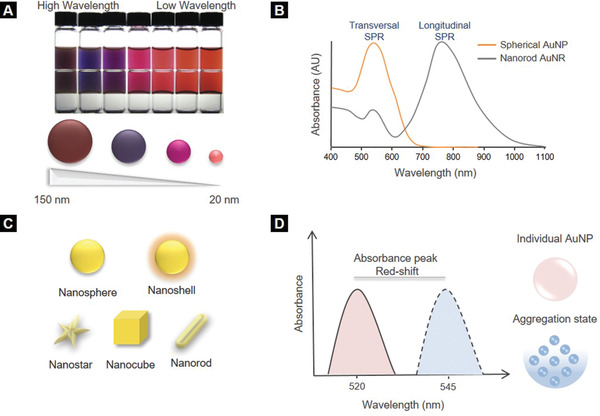
Optical properties of gold nanoparticles (AuNPs). A) Size‐dependent optical properties of AuNPs. As the mean diameter of AuNPs decreases from 150 to 20 nm, the peak absorbance shifts to a lower wavelength, resulting in a brighter color of the AuNPs solution. B) Absorption profiles of two AuNPs (40 nm size) of different shape corresponding to transverse (≈520 nm) and longitudinal (near the infrared region of spectrum) surface plasmon resonances, showing the shape dependent optical properties of AuNPs. C) Different shapes of AuNPs. D) Aggregation‐dependent optical properties of AuNPs. Aggregation of AuNPs causes peak absorbance shift to a higher wavelength.

Within the field of nanotechnology, AuNPs are extensively used and are particularly attractive in diagnostics due to their facile chemical and tunable optical properties. The remarkable optical performance of AuNPs originates from their unique interaction with light. The collective oscillation of electrons on AuNPs surface, known as localized surface plasmon resonance (LSPR), leads to a powerful extinction of light.^[^ ^27^ ^]^ The LSPR phenomenon is highly dependent on the size, shape, surface chemistry, and aggregation state of AuNPs (**Figure** **2**). For instance, spherical AuNPs with a mean diameter ranging from 20 to 100 nm show a maximum absorbance from 520 to 570 nm, respectively, whereas those with sizes above 100 nm exhibit broader absorbance peaks.^[^ ^28^ ^]^ Apart from size, shape also plays a crucial, with gold nanorods and nanostars being particularly attractive due to their peak absorbance in the infra‐red region of the spectra (Figure 2B,C).^[^ ^28^ ^]^ Moreover, upon aggregation, AuNPs show a redshift in maximum absorption that can produce a color change in the solution in which they are dispersed in (Figure 2D). The fact that the optical properties of AuNPs can be easily tuned by changing their physicochemical properties enables their exploitation for diagnostic applications. Furthermore, their ease of functionalization with targeting probes makes them ideal biosensors for the detection of infectious agents and other biomolecules.^[^ ^29^ ^]^

In view of the above, AuNP‐enabled colorimetric biosensing of pathogens is among the most attractive applications. Mirkin et al. introduced a novel sensing strategy to identify DNA sequences upon the self‐assembly of AuNPs.^[^ ^30^ ^]^ Non‐complementary DNA oligonucleotides were attached to the surface of AuNPs (13 nm in size). Once a duplex DNA, complementary to the DNA oligonucleotides attached to the AuNPs, was added to the solution, NPs self‐assembled into aggregates. The interaction of capped‐AuNPs with DNA induced a color change in the solution, which could be tailored by varying the NPs size and the oligonucleotide sequence and length.^[^ ^30^ ^]^ This study opened up a new pathway of DNA‐NP hybrid materials with unique and tunable optical properties. Inspired by the above strategy, Elghanian et al. proposed a colorimetric method to selectively detect specific polynucleotides using mercaptoalkyloligonucleotide‐functionalized AuNP (13 nm) probes.^[^ ^31^ ^]^ Binding of AuNP probes to specific targeted DNA sequences resulted in a distinct shift in AuNP SPR peak. Despite the simplicity of this strategy, the method is restricted by its relatively low limit of detection (LOD: 10 fmol of target DNA). The above studies catalyzed the emergence of the future generation NP‐based platforms for the fast and sensitive colorimetric detection of pathogenic DNA in sepsis.

**Figure 3:**
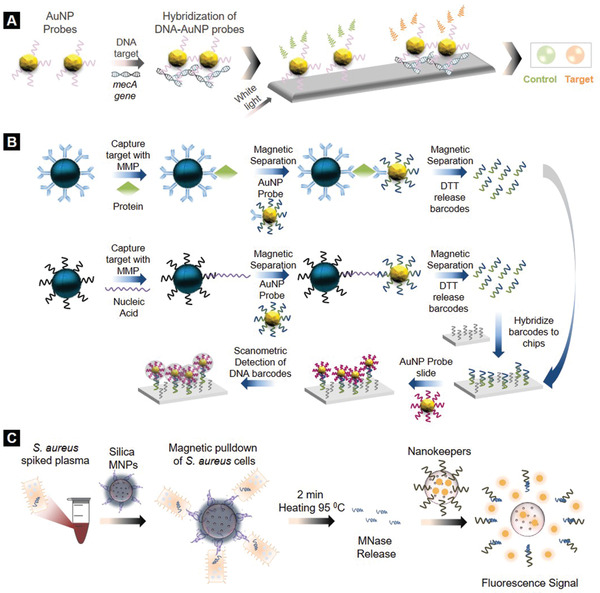
Nanodiagnostic technologies for sepsis using gold (AuNPs) and fluorescent nanoparticles. A) AuNP‐enabled colorimetric detection of DNA sequences. B) Bio‐barcode assay for specific DNA and protein detection. Reproduced with permission.^[^^34]^ Copyright 2006, Springer Nature. C) *S. aureus* biosensing utilizing the fluorescent properties of silica nanoparticles.

In a subsequent study, Storhoff et al. developed a “spot‐and‐read” colorimetric method using DNA‐modified AuNPs (50 nm) to rapidly detect specific *mecA* gene sequences of methicillin‐resistant *S. aureus* strains (**Figure** **3**A).^[^ ^32^ ^]^ Authors hypothesized that the use of bigger AuNPs (50 nm) would result in higher sensitivities compared to smaller AuNPs (13 nm), which were used in previous similar studies.^[^ ^30^, ^31^ ^]^ Interestingly, this method showed higher sensitivity (333 zmol or 2 × 10^5^ target molecules) compared to previous studies^[^ ^31^, ^33^ ^]^ and enabled a detectable color change in the samples solutions within 2 h.^[^ ^32^ ^]^

In another study, Mirkin and Hill developed an in vitro ligand exchange bio‐barcode assay for the detection of nucleic acid and protein biomarkers within 9–10 h (Figure 3B).^[^ ^34^ ^]^ Magnetic microparticles (MMPs) and oligonucleotide‐functionalized 13 nm AuNPs probes, carrying thiolated single‐stranded barcodes, were mixed and formed a sandwich around the target of interest. Barcodes were then released from the sandwich structure and hybridized. The DNA barcodes were detected using oligonucleotide‐conjugated AuNP probes with high sensitivity for several protein (10^−18^ m) and nucleic acid (10^−19^ m) targets. Based on this bio‐barcode assay, Nanosphere, Inc. developed an FDA approved test, the “Verigene,” to detect pathogens. In Verigene, silver‐enhanced AuNPs (13–20 nm) allow the qualitative identification of Gram‐positive bacteria and genes associated with bacterial infection.^[^ ^35^ ^]^ Each NP is functionalized with a defined number of oligonucleotides, specific to a particular protein of interest. High specificity, amplified signal readouts, stability and reduced toxicity are the key assets that led this assay to scalability and clinical applicability.

A rapid strain‐specific detection method was reported by Wang et al. using vancomycin‐conjugated magnetic beads and nucleotide‐labeled AuNPs probes.^[^ ^36^ ^]^ Conjugated magnetic beads were used to capture bacteria, whereas labeled AuNPs were designed to sense and detect three different bacterial types (*E. coli*, *K. pneumoniae*, and *S. aureus*) by hybridization‐induced color change. Bacterial samples were coincubated with magnetic beads in an integrated microfluidic device to allow capturing. Addition of strain‐specific AuNPs probes led to bacterial DNA hybridization, inducing thus color change in the contaminated sample. Interestingly, the investigated bacterial strains were detected within 25 min with capturing rates above 90%. Despite the promising results of this technology, the selectivity of the described microfluidic chip in the presence of more pathogens should be further explored.

More recently, a naked‐eye detection method of urease‐positive bacteria using magnetic beads and plasmonic AuNP sensors was proposed.^[^ ^37^ ^]^ Following magnetic capturing of bacteria and urea addition in solution, the pH‐dependent assembly of AuNPs induced red‐ or blue‐colored NP suspensions, reflecting the presence or not, respectively, of urease‐positive bacteria. As urease‐negative bacteria did not increase the pH upon urea addition, the acidic conditions of the solution led to AuNPs clustering and a blue colored test. Conversely, urease‐positive bacteria induced a rise in the pH of the solution due to NH_3_ production and prevented AuNPs clustering. The developed strategy enabled the ultrasensitive (10 CFU mL^−1^, colony forming unit mL^−1^) detection of *Proteus mirabilis* in human urine samples within 40 min.

In a later study of the same group, carboxylate‐, amine‐ and polyvinylpyrrolidone‐coated AuNP probes were exploited for the rapid detection of interleukin‐6 (IL‐6) using a smartphone‐based colorimetric device.^[^ ^38^ ^]^ The developed nanoplatform entailed a paper‐based biosensor coupled with a smartphone app for colorimetric signal quantification. AuNP probes were immobilized onto the filter paper and the generated color was assessed with the custom designed mobile app. The NP‐enabled mobile biosensor enabled the sensitive detection of IL‐6 in buffer solution and IL‐6‐spiked blood with 0.1 and 12.5 pg mL^−1^ LOD, respectively, within 17 min.

A novel point‐of‐care device inspired by lens‐free interferometric microscopy (LIM) which encompasses a plasmonic Au nanohole substrate and custom bioprinted microarrays was proposed by Dey et al.^[^ ^39^ ^]^ Upon the incubation of plasma samples from healthy donors, noninfectious systemic inflammation controls and sepsis patients onto the Au nanohole substrate, authors were able to optically detect *E. coli* with an LOD of 400 CFU mL^−1^. The LIM device was further developed to separately sense CRP, IL‐6, and miRNA‐16 biomarkers in spiked PBS samples.^[^ ^40^ ^]^ Specific antibodies for the targets of interest were immobilized to the Au nanohole array chips and CRP, IL‐6, and miRNA‐16 markers were quantified by the photonic biosensor with LOD of 18, 88, and 6 µg mL^−1^, respectively.

Novel diffusometric DNA nanosensors, composed of 200 nm fluorescent polystyrene beads sandwiched with methicillin‐resistant *S. aureus* and 80 nm AuNPs oligonucleotide probes, were designed by Wang et al. to capture and amplify respectively *S. aureus* DNA.^[^ ^41^ ^]^ The sensing mechanism was based on the NP size‐dependent Brownian motion, by which any changes in NPs diameter could be reflected on diffusivity and measured. In the presence of bacterial DNA, the size of PS nanobeads increased, leading to a decrease in their Brownian motion and thus lower diffusivity. The diffusometric DNA nanosensor allowed *S. aureus* DNA quantification with 10 × 10^−12^ m LOD.

### 2.2 Fluorescent Nanoparticle‐Enabled Sepsis Diagnosis

Fluorescence‐based techniques are commonly applied for the detection of pathogen‐related molecules in microbial infections. Owing to their unique fluorescent properties and superior photostability over conventional fluorophores, fluorescent NPs, such as QDs and silica NPs, enhance detection sensitivities. ^[^ ^22^, ^42^ ^]^

**Figure 4:**
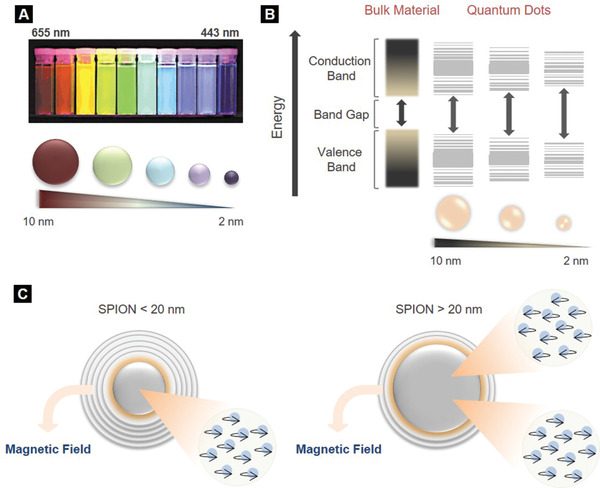
Size‐dependent properties of quantum dots (QDs) and SPIONs. A) Size‐dependent optical properties of QDs. As the mean diameter of QDs decreases from 10 to 2 nm, emission maxima shift to smaller peak wavelengths (from 655 to 443 nm), resulting in a color change of the emitted light from red to blue. Reproduced with permission.^[43]^ Copyright 2015, American Chemical Society. B) Splitting of energy levels in quantum dots due to the quantum confinement effect. Bandgap increases with decrease in size of the nanocrystal. Depending on the size of NPs, the bandgap, which is between the highest valence band and the lowest conduction band, fluctuates leading to color shifts from blue to red in the emitted light. Smaller QDs emit blue light, whereas bigger ones red. C) Size‐dependent magnetic properties of SPIONs. The electrons of SPIONs with a mean diameter lower than 20 nm spin in the same direction, while SPIONs with a higher diameter have multiple domains of electrons which spin in opposite directions. Reproduced with permission.^11]^ Copyright 2010, Massachusetts Medical Society.

QDs are semiconducting nanocrystals with their size ranging between 2 and 10 nm and determining the color of the emitted light (**Figure** **4**A). Their size‐dependent properties stem from the quantum confinement effect (Figure 4B), which leads to the production of various emission wavelengths. This correlation between the size and the energy levels of QDs allows their tunable manufacturing for a variety of applications, including bioimaging.^[^ ^43^ ^]^ Furthermore, their unique electrical and optical properties make them superior to conventional fluorophores. QDs have been observed to exhibit longer fluorescent lifetimes than traditional fluorophores,^[^ ^44^ ^]^ resulting in improved sensitivities and signal readouts.^[^ ^45^ ^]^ Silica NPs can be used in various biological applications owing to their excellent biocompatibility, thermal stability and low cytotoxicity. The development of mesoporous silica NPs in particular, with an intermediate pore size range between 2 and 50 nm, has catalyzed the evolution of new diagnostic possibilities. Their increased image contrast, chemical stability, and controllable size with a narrow distribution and their ability to conjugate with functional moieties within the pores have proven extremely beneficial for bioimaging and biosensing.^[^ ^46^, ^47^ ^]^ Herein, we review some examples of QD‐ and silica‐based sensors for pathogen detection in sepsis.

For instance, mannose‐modified fluorescent 3 nm carbon QDs (Man‐CQDs) were synthesized by Weng et al. to label *E. coli*.^[^ ^48^ ^]^ Bacteria were coincubated with Man‐CQDs for 1 h and samples were fluorescently characterized. Selective binding of Man‐CQDs to *E. coli* resulted in the emission of bright blue fluorescence indicating the presence of the pathogen with a LOD of roughly 450 CFU mL^−1^. The selectivity of this nanoplatform toward *E. coli* was attributed to the specific interaction of mannose units with the FimH lectin of *E. coli*. Subsequently, human urine samples spiked with *E. coli* were incubated with Man‐CQDs. The photostability of the fabricated formulation and the unique fluorescent properties of QDs enabled the successful labeling and detection of *E. coli* in all samples with a minimum detectable concentration of ≈10^3^ CFU mL^−1^. Even though, the proposed method is promising, further optimization is required in order to achieve higher sensitivities and detect pathogens in much lower concentrations.

Recently, green and red emitting QDs (CdSe‐QDs) were employed to allow the simultaneous labeling and quantification of CRP and IL‐6 biomarkers by a point‐of‐care lateral flow assay (LFA).^[^ ^49^ ^]^ A customized software tool, the MultiFlow‐Shiny app, was used to process and analyze the LFA experimental data. By a single UV‐light source, both CRP and IL‐6 were quantified with 42.5 × 10^−9^ and 0.21 × 10^−12^ m detection limits, respectively, values which are within the clinical range observed in sepsis.

Using antibody‐bioconjugated silica NPs (60 nm) encapsulated with fluorescent dye molecules, Zhao et al. successfully detected single bacterial cells within 20 min.^[^ ^50^ ^]^ Thousands of dye molecules were encapsulated in each silica NP and significantly contributed in signal amplification, enabling ultrasensitive quantitation of pathogenic targets. This method was subsequently employed to simultaneously detect multiple bacterial species (*E. coli*, *S. aureus*, and *S. typhimurium*).^[^ ^51^ ^]^ Multicolored silica NPs were conjugated to monoclonal targeted antibodies specific to these pathogens and facilitated multiple bacteria detection.

Similarly, a fast and sensitive assay for *S. aureus* detection was developed by Borsa et al. using aptamer‐functionalized silica MNPs (187 nm) and fluorophore‐loaded biosensors made from silica NPs, called “Nanokeepers,” specific to micrococcal nuclease (MNase) (Figure 3C).^[^ ^52^ ^]^ MNase is the most prevalent biomarker for *S. aureus*, as it is naturally secreted from bacterial cells. Blood samples spiked with 10^2^ CFU mL^−1^ *S. aureus* were incubated with functionalized silica MNPs to capture bacterial cells by magnetic pulldown. Approximately 61% of bacterial cells were successfully captured from whole blood. Subsequent heating led to MNase release into the solution, in which Nanokeepers were added. Nanokeepers significantly enhanced the fluorescence signal and enabled the sensitive detection of *S. aureus* in the samples. Interestingly, the fluorescence signal was amplified as the number of *S. aureus* cells was increasing (>10^5^ cells mL^−1^), while the LOD was calculated at 682 cells mL^−1^, which is promising and requires further investigation.

### 2.3 Magnetic Nanoparticle (MNP)‐Enabled Sepsis Diagnosis

A variety of innovative applications has been emerged utilizing MNPs. MNPs are far more susceptible to external magnetic fields than bulk materials and this stems from the higher number of electrons that spin in the same direction. Additionally, magnetic field strength is size‐dependent.^[^ ^11^ ^]^ For instance, iron oxide NPs smaller than 20 nm, known as superparamagnetic iron oxide NPs (SPIONs), have a single domain of electrons that spin in the same direction, while iron oxide particles with diameter greater than 20 nm have multiple domains of electrons that spin in opposite directions (Figure 4C). Therefore, SPIONs offer higher magnetic liability to external magnetic field than other paramagnetic materials.^[^ ^11^ ^]^ Another asset of SPIONs is demagnetization once the external magnetic field is removed, which is very important for biomedical applications. Several MNPs, especially SPIONs, have been FDA approved and are currently used as contrast agents for MRI,^[^ ^53^, ^54^ ^]^ and this makes them very attractive to assist in sepsis diagnosis.

**Figure 5:**
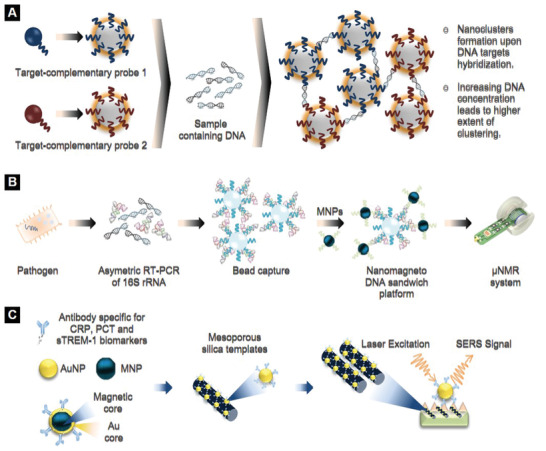
Nanodiagnostic technologies for sepsis using magnetic nanoparticles (MNPs). A) Schematic representation of T2MR diagnostic platform.Reproduced with permission.^[^ ^55^ ^]^ Copyright 2013, American Association for the Advancement of Science (AAAS). B) Nanomagneto‐DNA assay for the detection of bacterial 16S rRNA. Reproduced with permission.^[^ ^59^ ^]^ Copyright 2013, Springer Nature. C) SERS substrates to anchor antibody‐decorated gold‐coated MNPs for sepsis diagnosis.

In this context, Neely et al. developed a T2MR (T2 magnetic resonance) diagnostic platform using oligonucleotide probes for Candidemia decorated with SPIONs (800 nm).^[^ ^55^ ^]^ The SPION‐based biosensor enabled the ultrahigh sensitive (≈1–3 CFU mL^−1^) detection of five clinically common *Candida* species in whole blood within 3 h. SPIONs were covalently conjugated with oligonucleotides to generate two populations of probes, each of them carrying a target‐complementary probe (**Figure** **5**A). Blood spiked with *Candida* and unknown clinical samples were incubated with SPIONs. Hybridization of DNA targets led to the formation of SPION clusters with the clustering degree reflecting the DNA concentration. The amplified *Candida* DNA was then measured by PCR generating T2MR signals. Strikingly, the T2MR biosensor formed the basis for the design of an automated instrument platform. “T2Candida panel,” as the pathogen detection nanoplatform was later called, has been FDA approved, facilitating direct and rapid analysis of whole blood specimens for the identification of five *Candida* species without any requirement for blood culture. Results from the first extensive multicenter clinical trials of T2Candida panel demonstrated an overall specificity and sensitivity per patient of 98.1% and 91.0%, respectively, with an average time to species identification of 3 to 5 h.^[^ ^56^, ^57^ ^]^

Simplicity, low‐cost, single‐cell detection accuracy, minimum sample processing and fast assay time are crucial features for a powerful diagnostic tool with clinical potential. In respect of this, Issadore et al. developed a portable microfluidic chip‐based micro‐Hall (*μ*Hall) platform for robust and high‐throughput (10^7^ CFU/min) bacterial detection.^[^ ^58^ ^]^ Targeted bacteria were labeled by MNPs and were rapidly detected by the miniaturized *μ*Hall device.

In another study, Chung et al. described the design of 20 nm nanomagneto‐DNA probes to rapidly and sensitively profile various pathogens in clinical samples by targeting the bacterial 16S ribosomal RNA region (Figure 5B).^[^ ^59^ ^]^ Although this rRNA region is consistent between all bacteria, it is characterized by species‐associated variabilities in distinct areas of the genetic sequence and can therefore allow distinction between bacterial types.^[^ ^60^ ^]^ Combination of the oligonucleotide–MNP probes with a miniaturized micronuclear magnetic resonance system for signal readout^[^ ^61^ ^]^ enabled the accurate detection and phenotype of a large pool of 13 different bacterial species within 2 h with a 0.5 × 10^−12^ m LOD.^[^ ^59^ ^]^

A surface‐enhanced Raman scattering (SERS)‐based assay was developed by Nguyen et al. to monitor a triplex panel of sepsis protein biomarkers: CRP, PCT, and sTREM‐1 (Figure 5C).^[^ ^62^ ^]^ Mesoporous silica templates were synthesized with magnetic immune colloids to anchor 20 nm antibody‐decorated Au‐coated MNPs. Fabrication of these SERS substrates enhanced Raman signal and catalyzed the detection of CRP, PCT, and sTREM‐1 biomarkers in human serum samples with LOD values being relatively low at 27, 103, and 78 × 10^−12^ m for CRP, PCT, and sTREM‐1, respectively.

Another interesting SERS‐based strategy proposed the use of biomimetic octopus‐like NPs with a magnetic core and a decorated with aptamers polymeric multiarm shell to specifically capture and detect *S. aureus* among a pool of four pathogens.^[^ ^63^ ^]^ The polymeric arms and the multivalent ligands worked synergistically, imitating the suction cups of an octopus, to enhance bacterial attachment and capture with high sensitivity (10 CFU mL^−1^).

During sepsis, the stimulation of host immunity triggers the excessive production of reactive oxygen species (ROS) in the blood circulation and affected organs, and thus ROS have been alternatively considered as sepsis biomarkers. On the grounds of this, the clinically approved gadolinium‐diethylenetriamine penta‐acetic acid (Gd‐DTPA) with an hyaluronic acid (HA)‐decorated iron oxide core (SPIONs) was recently employed as a contrast agent to probe ROS by MRI in an lipopolysaccharide (LPS)‐induced sepsis mouse model.^[^ ^64^ ^]^ The unlimited tissue penetration depth of SPION nanoprobes accompanied with the HA‐triggered ROS degradation mechanism and the subsequent release of Gd‐DTPA enabled ultrasensitive (0.2 × 10^−6^ m) ROS imaging in vivo.

MNPs can also be used as affinity probes to selectively trap infectious agents and enrich their low concentration levels from a complex biological matrix. These nanoscale probes can enhance the sensitivity of proteomic techniques by eliminating the obstructive signal interference from other biomolecules.^[^ ^26^ ^]^ Recently, Hasan et al. employed MNPs (<15 nm) modified with 3‐aminopropyltriethoxysilane to allow interaction with *β*‐lactam antibiotic amoxicillin and effectively detect *S. aureus* and *E. coli* by matrix‐assisted laser desorption–ionization (MALDI‐MS).^[^ ^65^ ^]^ Penicillin binding proteins (PBPs) naturally contained in bacteria were bound to amoxicillin functionalized MNPs. Subsequently, MALDI‐MS was performed to comprehensively analyze the attached PBPs onto the surface of MNPs. Both bacterial MALDI mass spectra were considerably enriched with PBPs, owing to the high affinity of amoxicillin engineered MNPs for *β*‐lactam. Noteworthy, the lowest detectable concentrations for both *S. aureus* and *E. coli* were ranging between 10^3^ and 10^4^ CFU mL^−1^.^[^ ^65^ ^]^

### 2.4 Liposome‐Enabled Sepsis Diagnosis

**Figure 6:**
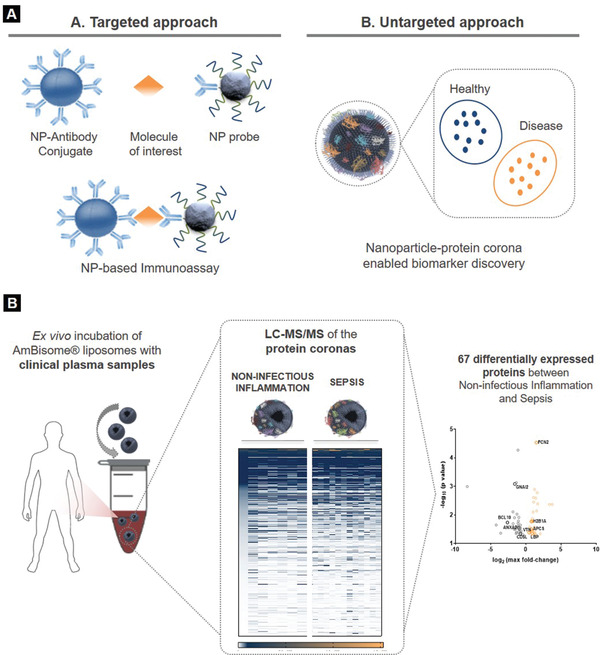
Nanodiagnostic technologies for sepsis using liposomes. A) NP‐assisted technologies to aid the biomarker development pipeline in sepsis. The targeted approach of NP‐based immunoassays for the detection of biomarkers A. and the untargeted approach of the NP–biomolecule corona for the discovery of biomarkers B. B) Schematic representation of the liposome–protein corona workflow for sepsis biomarkers enrichment.

Apart from boosting the signals of diagnostic assays targeting specific and already known biomolecules, NPs can be also used for the benefit of novel biomarkers discovery (**Figure** **6**A). Particularly interesting is the recently suggested concept of exploiting the NP–protein corona, a layer of proteins adsorbed onto NPs surface once in contact with biofluids, to harvest disease‐specific, previously unknown biomarker proteins by high throughput label‐free MS. Triggered by the initial notion of the “personalized protein corona”,^[^ ^66^ ^]^ the use of the NP–protein corona fingerprinting to differentiate between healthy and nonhealthy samples inspired a series of investigations.^[^ ^67^, ^68^, ^69^, ^70^, ^71^, ^72^ ^]^In one of these studies, polyethylene glycol functionalized liposomes were injected into the blood circulation of tumor‐bearing mice and subsequently recovered to characterize the in vivo formed protein corona by liquid chromatography‐MS (LC‐MS/MS). Authors demonstrated that the liposomes enabled the capture and amplification of low molecular weight and low abundant proteins from the blood circulation of tumor‐bearing mice, which could not be detected by conventional proteomics.

In the context of sepsis, Papafilippou et al. proposed the use of liposomes as blood sepsis‐specific protein scavengers (Figure 6B).^[^ ^70^ ^]^ Commercially available amphotericin B‐containing liposomes (AmBisome, 100 nm) were incubated with plasma samples obtained from sepsis and noninfectious acute systemic inflammation patients, and the resultant protein coronas were thoroughly compared by LC‐MS/MS. The proteomic comparison of liposome‐corona fingerprints revealed 67 differentially expressed proteins between sepsis and noninfectious acute systemic inflammation, with 9 out of these 67 being previously associated with bacterial infection pathways. This work provided evidence that NP‐enabled MS analysis can uncover panels of novel protein biomarkers for sepsis which would otherwise be undetectable.

## 3 Nanomonitoring for Sepsis Progression

Antimicrobial susceptibility assessment is crucial for the management of sepsis. The development of multidrug resistance (MDR) mechanisms by bacteria hinders antibiotic stewardship decision. Assays able to assess microbial sustainability antibiotic and efficacy can guide clinicians with treatment decision making and optimization of the antibiotic concentration, dose and administration frequency.^[^ ^73^ ^]^ In view of the above, several nano‐based technologies that can determine the antimicrobial susceptibility in real time have been developed. Here, we present some of the most relevant for sepsis.

**Figure 7:**
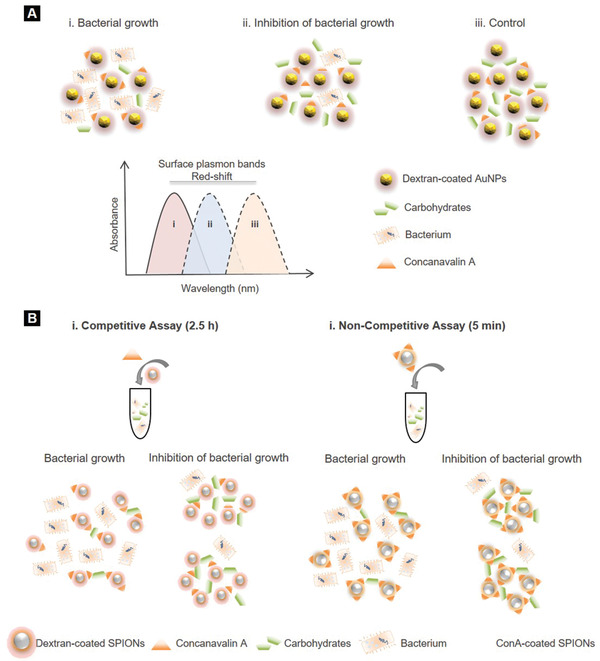
Nanomonitoring technologies for sepsis. A) Antimicrobial susceptibility assay using dextran‐coated AuNPs. In the presence of bacterial carbohydrate uptake i), the amount of free carbohydrates decreases, resulting in a decrease in the size of the gold nanoclusters, which corresponds to lower plasmon resonances. Under bacterial growth inhibition ii) or in sterile conditions iii), addition of Con A results in the formation of large nanoclusters and larger shifts in the surface plasmon band, owing to the presence of carbohydrates which have not been uptaken. Reproduced with permission.^[^ ^74^ ^]^ Copyright 2008, American Chemical Society. B) Bacterial metabolic activity assessment using SPION nanosensors and NMR‐based measurement of spin–spin relaxation time of the solution's water protons. Reproduced with permission.^[^ ^75^ ^]^ Copyright 2008, PLoS ONE.

Nath et al. reported an antimicrobial susceptibility assay using dextran‐coated AuNPs (25 nm) that formed nanoclusters in the presence of concanavalin A (Con A), a protein with high affinity to carbohydrates in bacterial suspension (**Figure** **7**A).^[^ ^74^ ^]^ In order to assess the bacterial metabolic activity, the surface plasmon bands of AuNPs were profiled following their incubation with *E. coli* (10^6^ CFU mL^−1^). Upon bacterial growth, carbohydrates were rapidly consumed and their amount in the medium decreased. Consequently, AuNPs formed small gold nanoclusters with lower plasmon resonances. Under bacterial growth inhibition, the presence of free carbohydrates, and thus Con A, induced the large self‐assembly of AuNPs, resulting in a significant redshift of the NPs surface plasmon band. This nanoplatform enabled the sensitive assessment of bacterial proliferation within 3 h. However, as microorganisms in most of sepsis cases are present in the blood of infected patients, testing bacterial susceptibility in blood‐ or urine‐containing media rather than bacterial media would be more representative of the real‐life conditions system.

This idea was further investigated by Kaittanis et al.^[^ ^75^ ^]^ who evaluated the bacterial metabolic activity and antimicrobial susceptibility in blood even at low populations (10^2^–10^4^ CFU mL^−1^) via water relaxation using either dextran‐ or Con A‐conjugated SPION‐based nanosensors (Figure 7B). At low bacterial growth and minimal metabolic activity, polysaccharides availability induced the formation of large nanoclusters leading to considerable change in spin–spin relaxation time of the solution's water protons. The consumption rate of nutrients was measured within 2.5 h and 5 min by dextran‐ and CoA‐coated SPIONs, respectively.^[^ ^75^ ^]^ This NMR‐based approach enabled rapid profiling of bacterial responses and eliminated the issue of strong media absorbance which is often observed in optical‐based assays.

Evaluating immune system responses could also enable sepsis progression monitoring. Using the FDA‐approved SPION‐based contrast agents “Feridex,” Wong et al. monitored the immune system activity in vitro and in vivo and particularly Kupffer cells.^[^ ^76^ ^]^ Once Kupffer cells sense an ongoing infection, they rapidly multiply. Meanwhile, the dominant mechanism by which Feridex is cleared from the body is phagocytosis by Kupffer cells.^[^ ^77^ ^]^ It was thus hypothesized that high Feridex uptake activity would reflect high levels of Kupffer cells, indicating an alert immune system due to potential infection.^[^ ^76^ ^]^ Mapping of Feridex NPs uptake by MRI in *E. coli* derived LPS‐induced murine monocytes and sepsis mouse models revealed the approximate amount of released Kupffer cells. LPS‐treated cells displayed a higher NP uptake than non‐LPS cells. Moreover, higher levels of iron internalization in the LPS‐treated monocytes compared to the untreated indicated an accelerated phagocytic activity. Although significant differences were observed in Feridex uptake in vitro, no differences were shown in vivo. However, this was a pilot study and further work is required to explore the role of Kupffer cells in sepsis.

## 4 Nanotreatment Technologies for Sepsis

Once sepsis is diagnosed, antibiotic therapy needs to be immediately initiated. However, sepsis treatment is extremely challenging, due to diagnostic doubts about the causative microbe.^[^ ^15^ ^]^ Considering the lengthy process of laboratory cultures, clinicians often prescribe a combination of antibiotics before the detection of the infection‐causing organisms, which catalyzes the massive healthcare issue of MDR strains. Even when the right antibiotic treatment is initiated, successful drug delivery is not always guaranteed, due to the poor pharmacokinetic properties of the administered agents.^[^ ^8^ ^]^

During sepsis, several pathophysiological changes occur which may alter drug pharmacokinetics. These can involve augmented renal clearance, leaky capillaries, impaired tissue penetration and renal clearance, hepatic dysfunction, changes in the volume of distribution (Vd), fluid shifts, drug absorption, drug metabolism and alterations in protein binding.^[^ ^78^, ^79^, ^80^, ^81^ ^]^ The release of pathogenic toxins in sepsis causes endothelial damage, increases capillary leakage, microvascular failure and compromises tissue perfusion, which in turn greatly affects drug distribution. The Vd of hydrophilic antimicrobials thus rises, resulting in lower plasma and tissue antimicrobial concentrations.

Drug‐induced toxicity is another issue that hampers antibiotics use.^[^ ^17^ ^]^ The low plasma levels of albumin in sepsis result in increased unbound fraction of drug which may potentially cause adverse side effects and toxicity.^[^ ^80^ ^]^ Furthermore, elevated risks of toxicity can be generated by decreased clearance and metabolism of drugs, due to renal failure and hepatic dysfunction, implications which are both often encountered in sepsis.^[^ ^79^ ^]^ Finally, poor blood perfusion to the peripheries and the disrupted microcirculation within organs during sepsis adversely compromises the systemic absorption of antibiotics.^[^ ^79^ ^]^ Consequently, antibiotics cannot always adequately treat sepsis disease and additional therapeutic agents are needed to either amplify antimicrobial activities or suppress any hyperactivity of the immune system.^[^ ^8^ ^]^

Nanoscale drug delivery platforms have proven to enhance the blood circulation time of antimicrobial agents, overcome the predominant issue of underdosing and minimize the arising adverse side effects.^[^ ^17^, ^82^, ^83^ ^]^ Additionally, alternative antisepsis “nano”therapeutic strategies have been explored using NPs to enable a) lipopolysaccharide (LPS) neutralization, b) blood purification from inflammatory mediators, pathogens, and endotoxins, c) Toll‐like receptors inhibition (TLR), and d) immune system modulation (immunomodulatory NPs). In the following section, we present the recent advancements of nanomedicine to combat sepsis disease.

### 4.1 Drug Delivery Nanoplatforms in Sepsis

The employment of NPs to encapsulate antimicrobial agents has contributed in addressing the issues of poor pharmacokinetics and toxicity. NPs can be engineered to exhibit improved solubility, biocompatibility, and pharmacokinetic profiles, while their functionalization with biological moieties enables stimulated activation for targeted antibiotic delivery. Moreover, nanocarriers facilitate prolonged systemic circulation and drug half‐life, as well as sustained drug release, enhancing thus therapeutic efficacy and minimizing systemic toxicity.^[^ ^84^, ^85^ ^]^

The FDA‐approved NP‐based AmBisome, Abelcet, and Amphotec, which are different liposomal formulations of the same antifungal agent amphotericin B, are some examples of commercially available drug delivery nanosystems (**Table** **3**). The clinical use of conventional amphotericin B has been previously restricted due to its substantial toxicity at a dose‐dependent manner.^[^ ^86^ ^]^ Its incorporation into a lipid bilayer has fueled the development of a novel antifungal drug which is safely administered to patients suffering from fungal infections. Amphotericin B is preferentially retained within the lipid bilayer until the moment of exposure to the fungus inducing thus minimum toxicity to the body.^[^ ^87^ ^]^ Apart from liposomes, other nanoplatforms, such as the PEGylated form of filgrastim (Neulasta), metal NPs, lipid nanocrystals, and virosome‐based vaccines, have also been developed for sepsis and bacterial infections treatment and are currently used in the clinic (Table 3).

**Table 3:** Commercially available nanotechnology‐based products for sepsis diagnosis and treatment

Purpose	Name	Company	Nanocomposition	Application	Clinical stage	Ref.
Diagnosis	Verigene test	Nanosphere Inc.	Oligonucleotide‐conjugated AuNPs	Bacterial infection	Commercially available	^[^ ^35^ ^]^
	T2 Candida	T2 Biosystems	Oligonucleotide‐conjugated SPIONs	Blood detection for Candidemia	Clinical trial	^[^ ^55^, ^56^ ^]^
	AbioSCOPE	Abionic SA	Nanofluidic technology	Sepsis diagnostic	Clinically validated and CE marked	^[^ ^173^, ^188^, ^189^, ^190^ ^]^
	IVD Capsule PSP	Abionic SA	Nanofluidic biosensors	Quantifies PSP concentration	Clinically validated and CE marked	^[^ ^173^, ^174^, ^188^ ^]^
Treatment	Abelcet	Enzon Pharmaceutical (Sigma‐Tau Pharmaceuticals)	Liposomal amphotericin B	Fungal infection	Commercially available	^[^ ^25^ ^]^
	AmBisome	Gilead Sciences	Liposomal amphotericin B	Fungal infection	Commercially available	^[^ ^87^, ^189^ ^]^
	Amphotec	Sequus Pharmaceuticals	Liposomal amphotericin B	Fungal infection	Commercially available	^[^ ^25^ ^]^
	Fungisome	Lifecare Innovations	Liposomal amphotericin B	Fungal infection	Commercially available	^[^ ^26^ ^]^
	Neulasta	Amgen Inc.	Filgrastim‐bound polymeric NPs	Fibrile neutropenia	Commercially available	^[^ ^17^, ^191^ ^]^
	LogiCath AgTive	Smiths Medical International	Nanosilver	Antimicrobial coating device	Commercially available	^[^ ^192^ ^]^
	PerOssal	Aap Impantate	Calcium sulfate and nanoparticulate hydroxyapatite Composite	Antibiotic delivery	Commercially available	^[^ ^193^ ^]^
	Spi‐Argent	Spire Biomedical Corporation	Nanosilver	Antimicrobial coating device	Commercially available	^[^ ^194^, ^195^, ^196^ ^]^
	TAK‐242	Takeda Global Research & Development Center, Inc.	Resatorvid emulsion	Sepsis	Commercially available	^[^ ^197^, ^198^, ^199^ ^]^
	PEV7	Pevion Biotech Ltd	r‐SAP2 virosomal vaccine	Recurrent vulvovaginal candidiasis	Commercially available	^[^ ^200^, ^201^ ^]^
	Cytosorb	CytoSorbents Corporation	Polymeric nanobeads	Hemoadsorption device for septic shock	Commercially available	^[^ ^175^, ^177^, ^178^, ^179^, ^180^ ^]^
	MAT2501	Matinas Biopharm	Amikacin‐loaded lipid nanocrystals	Bacterial infection	Commercially available	^[^ ^202^ ^]^

Moreover, there is a class of NPs, metal and metal oxide NPs, such as silver (AgNPs), zinc oxide (ZnO), copper oxide (CuO), titanium oxide (TiO_2_), and aluminum oxide (Al_2_O_3_) that possess inherit antimicrobial properties.^[^ ^84^ ^]^ AgNPs in particular, often referred to as “Nanoantibiotics,”^[^ ^85^ ^]^ serve as antibacterial agents against a plethora of Gram‐negative and positive bacteria, as well as drug resistant pathogens. The mechanisms behind AgNPs bactericidal activity still remain unclear. A characteristic ROS stimulating process and interactions between Ag molecules and the bacterial cell membrane, DNA and proteins have been suggested as possible mechanisms.^[^ ^26^ ^]^ Similar to AgNPs, the antimicrobial properties of ZnO, CuO, TiO_2_, and Al_2_O_3_ NPs derive from a photocatalytic production of ROS and involve the disruption of the bacterial membrane integrity, the obstruction of energy transduction and transport processes, as well as the attenuation of respiratory enzyme activity and DNA synthesis.^[^ ^26^ ^]^ Despite that metal and metal oxide NPs antimicrobial behavior is quite promising for sepsis treatment, research at the moment is focused on using them to combat other infectious diseases and not sepsis. This is out of the scope of this review; nevertheless, it is worth mentioning that such NP‐based systems could yield positive therapeutic outcomes in sepsis upon further investigation.

NP‐based antimicrobial delivery has also demonstrated to be able to overcome the predominant issues of biofilms and intracellular microbes. Biofilm‐forming bacteria are characterized by a rigid structure which obstructs the entrance of antimicrobial agents.^[^ ^88^ ^]^ The encapsulation of antibiotics with lipid‐ and polymer‐based NPs for instance has shown to act as a protective shield from enzymes and to improve antimicrobial efficacies against biofilm‐forming bacteria.^[^ ^89^ ^]^ Simultaneously, the small size of such NPs allows their entrance into host cells, facilitating drug transport and subsequent drug release at the desired spot.^[^ ^90^ ^]^ Poly(vinyl alcohol) (PVA)‐coated poly(lactide‐*co*‐glycolide) (PLGA) NPs containing the antimicrobial peptide esculentin‐1a were explored by Casciaro et al. to treat *Pseudomonas aeruginosa* lung infection.^[^ ^91^ ^]^ Their neutral hydrophilic surface favored their permeability through pulmonary mucus and intrapulmonary bacterial biofilm. The administration of esculentin‐1a‐loaded PVA‐PLGA NPs to *P. aeruginosa*‐infected mice decreased the pulmonary bacterial burden by 3 logs within 36 h compared to PBS‐treated controls and resulted in a 17‐fold stronger antimicrobial activity compared to free esculentin‐1a.^[^ ^91^ ^]^

In another study by Saude et al. an antimicrobial peptide, clavanin, was encapsulated in methacrylate polymeric nanocarriers between 120 and 372 nm to improve antibiotic and immunomodulatory effects in bacterial sepsis.^[^ ^92^ ^]^ In order to assess the in vitro antimicrobial activity of clavanin nanoplatform, Gram‐positive (*S. aureus*) and negative (*E. coli*, *K. pneumoniae*, and *P. aeruginosa*) colonies were incubated with free and NP‐encapsulated clavanin. The in vivo antibacterial activity was investigated by injecting free and NP‐encapsulated clavanin to two groups of mice with different sepsis severity levels. In vitro assays showed 91% inhibition of *S. aureus* development, but low bactericidal activity against the rest of bacteria. In septicemic mice, 100% and 40% survival rates were observed in sublethal (40–60% mortality) and lethal (90–100% mortality) groups, respectively. Even though clavanin nanoencapsulation enhanced antibiotic properties, the study is limited by the fact that animals were pretreated with the nanocarrier and were infected 15 min post‐treatment.

Another popular strategy to improve the pharmacokinetics of antibiotics is to engineer targeted NP‐based drug delivery systems. Surface modification of NPs with molecules that selectively bind to specific receptors onto bacterial walls can enhance the accumulation of a higher dose of antibiotic at the desired spot and minimize toxicity.^[^ ^26^ ^]^ Chono et al. encapsulated ciprofloxacin in engineered mannosylated liposomes for the treatment of respiratory bacterial infection.^[^ ^93^ ^]^ The developed nanoplatform exhibited increased selectivity to alveolar macrophages (MACs) after pulmonary administration in rats. Further pharmacokinetic and pharmacodynamic analysis revealed efficient antibacterial effects even at lower antibiotic doses than those used in the clinic.

**Figure 8:**
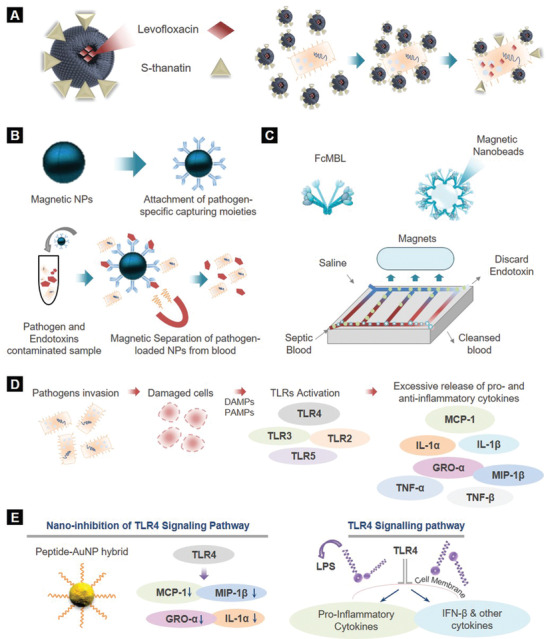
Nanotreatment technologies for sepsis. A) Levofloxacin encapsulation in S‐thanatin functionalized liposomes to target *K. pneumoniae*. B) Principle of magnetic separation‐based blood purification from endotoxins and pathogens. C) Extracorporeal blood cleansing device for sepsis treatment. Generic opsonin FcMBL is coated onto magnetic nanobeads to produce magnetic opsonins, which function as magnets. D) Representative flow chart of TLRs activation. Upon invasion of pathogens to the host body, TLRs activation is triggered from damaged cells and tissues (PAMPs and DAMPs). During sepsis, the dysregulated host response leads to dysregulated TLRs activation and thus excessive release of pro‐ and antiinflammatory cytokines. E) TLR4 signaling inhibition utilizing peptide AuNP hybrids.

In another study, Fan et al. designed S‐thanatin (Ts)‐functionalized levofloxacin (LEV)‐encapsulated liposomes (LPs) to target and eliminate clinical MDR isolates of *K. pneumoniae* in vitro and in vivo (**Figure** **8**A).^[^ ^94^ ^]^ Ts is a novel antimicrobial peptide that exhibits selective antimicrobial activity independent of current drug resistance and has a binding affinity to LPS.^[^ ^95^, ^96^ ^]^ LPs are known to serve as carriers for antibiotics in order to improve pharmacokinetics.^[^ ^97^, ^98^, ^99^ ^]^ Free antimicrobial LEV, LPs‐LEV, and Ts‐LPs‐LEV platforms were incubated with bacteria for 16–24 h. The application of LPs as drug carriers greatly improved the accumulation of LEV at the desired spot. Simultaneously, Ts enhanced targeting capacity and perturbed the lipid membrane bilayers of bacteria, which consequently catalyzed membrane permeability of LPs‐LEV and strong antibacterial activity. In a septic shock mouse model, Ts incorporation resulted in superior bacterial clearance in blood to free LEV and LPs‐LEV and in increased survival rate with 93.3% of treated animals surviving at 72 h.^[^ ^94^ ^]^

Yunus Basha et al. conjugated cyclodextrin‐complexed rifampicin and LEV to curdlan‐based NPs to accomplish elimination of *Mycobacterium smegmatis* macrophages (MACs).^[^ ^100^ ^]^ Curdlan is a linear glucan, known for its immunomodulatory and antimicrobial properties and it is recognized by dectin‐1 receptor expression in MACs.^[^ ^101^ ^]^ It was revealed that the dual nanocarrier was efficiently internalized by MACs with 1.8‐fold higher rates than that of fibroblasts and enabled the eradication of more than 90% of intramacrophage bacteria within 4 h.^[^ ^100^ ^]^ However, no significant difference was observed in the intracellular antibacterial activity between the nanocarrier and free drugs at 24 h, as at 24 h only 55% and 75% of rifampicin and LEV was released, respectively, rendering future optimizations essential.

Besides the use of targeting moieties, other strategies have been proposed to accomplish efficient antimicrobial delivery. Infectious microenvironments (IMEs) are known for their low pH, enzyme overexpression and bacterial toxins.^[^ ^102^ ^]^ The encountered acidic environment at IMEs, mainly induced by the metabolic processes of bacteria, contributes in the loss of antibiotic activity.^[^ ^103^ ^]^ The development of pH‐sensitive or/and enzyme‐responsive NPs that can act as shields at physiological conditions (pH = 7.4), but fuse within bacterial cells and release the antibiotic at low pH or/and the presence of specific enzymes has opened up a multitude of drug delivery possibilities.^[^ ^26^ ^]^

For example, Radovic‐Moreno et al. fabricated vancomycin‐encapsulated pH‐responsive and surface charge‐switching poly(d,l‐lactic‐*co*‐glycolic acid)‐*b*‐poly(l‐histidine)‐*b*‐poly(ethylene glycol) (PLGA‐PLH‐PEG) NPs to treat bacterial infections.^[^ ^103^ ^]^ Under acidic conditions, due to the positive surface charge of the poly(l‐histidine) component, the PLGA‐PLH‐PEG nanocarrier yielded overall positive *ζ*‐potentials, facilitating strong electrostatic‐mediated binding to the bacterial wall of *S. aureus* and *E. coli*. Interestingly, the incorporation of vancomycin within the polymeric nanoplatform resulted in a higher minimum inhibitory concentration than that of the free drug and in significantly enhanced activity at pH = 6 compared with the free vancomycin.^[^ ^103^ ^]^

More recently, Zhang et al. synthesized polymeric micelles coated with intercellular adhesion molecule‐1 (ICAM‐1) antibodies to codeliver ciprofloxacin and an anti‐inflammatory agent (2‐[(aminocarbonyl)amino]‐5‐(4‐fluorophenyl)‐3‐thiophenecarboxamide) to the IMEs of peritonitis‐induced sepsis mice.^[^ ^104^ ^]^ Due to the ICAM‐1 coating, the developed nanoplatform successfully targeted the inflamed endothelium and bound to bacteria in vitro. Simultaneously, the low pH and the bacterial enzymes present in the IME triggered efficient drug release from the pH sensitive and enzyme responsive polymeric NPs at the infection site. Strikingly, 90% of mice survived at 50 h following treatment with the bioresponsive nanoplatform, while bacterial burden in blood, leukocyte numbers and cytokines levels (TNF‐*α*, IL‐6, and IL‐1*β*) significantly reduced compared to the controls and free drug.^[^ ^104^ ^]^

Apart from taking advantage of the acidic environment of the infectious site, the secretion of specific biomolecules, such as bacterial toxins and enzymes can be also exploited for efficient antibiotic delivery. Pornpattananangkul et al. developed vancomycin‐encapsulated liposomes, coated with chitosan‐modified AuNPs, which allowed the smart release of drug at the infectious spot once they encountered bacterial toxins.^[^ ^105^ ^]^ The functionalization of liposomes with chitosan‐coated AuNPs protected them against uncontrollable fusion with one another or with bacterial membranes, preventing thus antibiotic leakage. This smart coating enabled only the access of pore‐forming proteins, such as bacterial toxins, to the liposomes for controlled drug release. Once liposomes were incubated with *S. aureus*‐secreted toxins, antibiotic leakage through the created by toxins pores was triggered with 100% of the encapsulated drug being released, inhibiting thus bacterial growth within 24 h.^[^ ^105^ ^]^

On a similar note, a lipase‐sensitive vancomycin‐encapsulated polymeric triple‐layered nanogel was designed by Xiong et al. to achieve efficient and selective drug release to the infectious spot and kill *S. aureus* bacteria.^[^ ^106^ ^]^ The rapid drug release mechanism of the nanogel was based on the poly(*ε*‐caprolactone) interlayer between the cross‐linked polyphosphoester and the PEG shell, which degraded once in contact with bacterial lipases. The developed nanoplatform delivered the drug efficiently into bacteria‐infected cells and led to significantly higher bacterial growth inhibition intracellularly than free vancomycin.^[^ ^106^ ^]^ This lipase‐sensitive polymeric nanogel was further optimized with mannose to selectively target MACs and facilitate antibiotics accumulation at bacterial infection sites through macrophage‐mediated transport.^[^ ^107^ ^]^ Strikingly, in vivo injection of mannosylated nanogels in bacteria‐infected zebrafish embryos significantly enhanced the therapeutic efficacy of vancomycin and resulted in higher survival rates than the free drug.

### 4.2 NP‐Enabled Alternative Methods to Combat Sepsis

As the identification of the infection source is not always feasible, alternative approaches, such as neutralization of pathogen‐released molecules and blood clearance from cytokines, pathogens, and their endotoxins, NP‐enabled cell therapies have been explored for sepsis therapy.^[^ ^108^, ^109^, ^110^, ^111^, ^112^, ^113^, ^114^, ^115^ ^]^

NP‐enabled LPS neutralization is one of the first alterative techniques investigated to aid in sepsis treatment. LPS is an endotoxin of Gram‐negative bacteria, anchored onto their outer membrane. Continuous exposure to LPS induces a deregulation of inflammatory cytokines in the bloodstream, known to trigger sepsis.^[^ ^116^ ^]^ Inhibition of LPS is a promising strategy for sepsis therapy and astonishingly, several nanostructures have been studied to serve this. For instance, Mishra developed polymeric capped nanostructures loaded with antimicrobial ciprofloxacin to specifically target LPS in vivo.^[^ ^108^ ^]^ Cytokines production (TNF‐*α* and NO) was significantly reduced upon administration of 4 µg mL^−1^ ciprofloxacin nanocarriers in LPS‐induced septic animals, suggesting that LPS was effectively targeted and inhibited. However, reduction of the administered dose led to insignificant changes in cytokines release, indicating the need for further optimization.

In another study, Mas‐Moruno et al. developed acyl nanostructural peptides to neutralize LPS activity.^[^ ^109^ ^]^ Peptides with longer acyl chains (C_16_) showed greater LPS‐neutralizing activity and lower cytotoxicity than the original acetylated peptides. Structural analysis by TEM revealed that N‐acylation with long chains promoted the formation of micellar and fibrillary‐like nanostructures, correlating high anti‐LPS activity with nanostructure formation.

Another approach to combat sepsis is cytokines removal from blood plasma. Pro‐ and antiinflammatory cytokines, such as TNF‐*α* and IL‐6, are released in response to infection during sepsis.^[^ ^117^, ^118^ ^]^ Cytokines are a double‐edged sword in sepsis, because on the one hand they are responsible to eliminate the infection, whereas on the other hand their excessive release can cause severe and irreparable organ damage.^[^ ^117^, ^118^ ^]^ The removal of inflammatory cytokines from the blood circulation of sepsis patients has been proposed as an efficient way to ease disease symptoms. The design of porous and magnetic NPs with high adsorption capacity have been widely explored to facilitate rapid and efficient clearance of blood from cytokines, owing to their unique structural and magnetic properties, respectively.^[^ ^110^, ^111^ ^]^

One indicative example is a study performed by Yachamaneni et al., who developed a series of porous carbide‐derived carbons (CDCs) to adsorb and remove cytokines from plasma samples.^[^ ^110^ ^]^ Porous CDCs of different surface chemistries and sizes were synthesized under various temperature and annealing conditions. Human plasma was spiked with cytokines (IL‐6, TNF‐*α*, and IL‐1*β*) at comparable concentrations with those in plasma of septic patients. ELISA experiments were performed to investigate the effect of CDCs synthetic identity on the removal of cytokines. Treatment with CDCs postannealed in Ar or NH_3_ and synthesized from <38 µm sized precursor particles facilitated the highest removal efficiency of all cytokines (99–100%) within 60 min. This study highlights the importance of NPs synthetic identify for efficient blood purification and fueled the design of other carbon‐based NPs,^[^ ^119^, ^120^ ^]^ such as graphene nanoplatelets,^[^ ^121^, ^122^ ^]^ with cytokines adsorption capacity.

The employment of nanomagnets is another popular strategy to purify blood from the inflammatory cytokines (Figure 8B).^[^ ^111^, ^123^, ^124^, ^125^ ^]^ Core/shell nanomagnets were functionalized with antihuman IL‐6 antibodies and were incubated with IL‐6 spiked human blood samples under gentle agitation.^[^ ^111^ ^]^ The target compound (IL‐6) was captured in vitro and removed from blood by magnetic pulldown. Purified blood was then analyzed by ELISA to measure any remaining toxin and determine the removal efficiency of the developed nanomagnets.

Apart from LPS neutralization and removal of inflammatory mediators from blood, another interesting approach is blood purification directly from bacteria and their endotoxins. Based on this concept, Lee et al. modified MNPs by PEG and zinc‐coordinated bis(dipicolylamine) (bis‐Zn‐DPA), a synthetic ligand that binds to bacteria, to efficiently remove *E. coli* from blood samples.^[^ ^112^ ^]^ MNPs_PEG‐bis‐Zn‐DPA_ was incubated with *E. coli* (10^7^ CFU mL^−1^)‐spiked blood samples and after two rounds of magnetic separation *E. coli* was completely eliminated from blood. Authors also developed a magnetic microfluidic single inlet‐dual outlet device with three magnets placed in series along the channel to purify blood. While this method enabled the selective and rapid removal of *E. coli* from blood, gradual accumulation of MNPs near the magnets reduced magnetic separation efficiency. Nevertheless, upon further optimization, nanodevices like this hold great potential for clinical translation.

Along similar lines, Kang et al. developed an extracorporeal blood cleansing device for sepsis therapy inspired by the microarchitecture of spleen, referred to as the “biospleen,” to continuously remove pathogens and toxins from blood without first identifying the infectious agent.^[^ ^113^ ^]^ A genetically engineered form of the human opsonin mannose‐binding lectin (FcMBL) that binds to a variety of pathogens was coated onto magnetic nanobeads to produce magnetic opsonins. As shown in Figure 8C, a high‐flow vascular channel was perfused with septic blood and interconnected by open slits to a parallel flow channel perfused with isotonic sterile saline. Magnetic opsonins were added to the flowing septic blood and passed through an incubation loop that promoted nanobead‐pathogen binding. Stationary magnets positioned above the channel, pulled the magnetic opsonins and bound pathogens through the open slits into the saline‐filled channel and into a discard collection vial. In vivo testing in a rat bacteremia model showed 90% depletion of live pathogen levels from blood within 1 h and significantly lowered blood levels of multiple proinflammatory cytokines. In a rat acute endotoxic shock model, substantial survival improvement, decrease in the LPS levels of organs and retrieval of physiological responses (temperature and respiratory rate) were observed after treatment with biospleen.^[^ ^113^ ^]^

In another study, Henry et al. engineered liposomes to isolate bacterial toxins produced during infection.^[^ ^114^ ^]^ Artificial liposomes, composed of cholesterol and shyngomyelin, were developed to promote binding to toxins released by a variety of pathogens. The binding of liposomes to toxins worked as the protection shield of cells against membrane damaging substances, which would otherwise inevitably cause cell lysis. The toxin‐sequestration mechanism of liposomes was also tested in vivo using mice infected by *S. pneumoniae* and *S. aureus*. Authors investigated whether complementary antibiotic treatment along with liposomes would boost the protective mechanism in mouse bacteremia models. Strikingly, the combination of liposomal toxin‐sequestration and antibiotics was more effective against sepsis than liposomes alone.

Another endotoxin neutralization strategy, based on a novel biohybrid motor system composed of living MAC cells and magnesium (Mg) microparticles was also presented by Zhang et al.^[^ ^126^ ^]^ In an in vitro LPS endotoxin experiment, Mg‐attached MACs bound to more LPS compared to free MACs, due to the propulsion behavior of the MAC‐Mg motors, which allowed the rapid movement and transport of MACs through the solution. The use of MAC‐Mg motor enabled 13% higher removal of endotoxin LPS than free MACs.

More recently, an interesting approach was proposed by Hou et al. by which MACs equipped with mRNA encoding the antimicrobial peptide IB367 and cathepsin B (AMP_IB367_‐CatB) were encapsulated in vitamin C lipid NPs to enable the elimination of *S. aureus* and *E. coli* in vivo.^[^ ^115^ ^]^ CatB was incorporated to transfer and enable the release of the AMP_IB367_ into lysosomes, while vitamin lipid NPs allowed the accumulation of AMP_IB367_‐CatB specifically in MAC lysosomes, a critical location for antimicrobial defense. The adoptive transfer of MACs containing AMP_IB367_ in the lysosomes significantly reduced the bacterial burden in the blood and major organs of *S. aureus*‐induced sepsis mice with immunosuppression within 24 h, and enabled restoration of body conditions within 72 h after treatment. In a mixed *S. aureus* and *E. coli*‐induced sepsis mouse model, the transfer of bone marrow‐derived MACs using the AMP_IB367_‐CatB nanoplatform resulted in a significantly reduced bacterial burden in blood and major organs within 24 h and in increased survival rates with 83% of treated animals surviving at 720 h.^[^ ^115^ ^]^

### 4.3 Nanoinhibition of Toll‐Like Receptors (TLR) Signaling

It is now well established that TLRs activation is triggered from damaged cells and tissues in response to the invasion of pathogens and endogenous molecules into our body as a defense mechanism.^[^ ^127^ ^]^ However, dysregulated TLR activation can disrupt the immune homeostasis and lead to an excessive release of pro‐ and antiinflammatory cytokines and chemokines (Figure 8D).^[^ ^128^ ^]^ As TLRs can recognize a variety of pathogen associated molecular patterns (PAMPs) and damage associated molecular patterns (DAMPs), it is critical to manipulate or inhibit their activity.^[^ ^129^ ^]^ Nanobased antagonists have been recently described as a promising strategy to modulate and suppress TLR signaling.^[^ ^127^ ^]^ These nanoinhibitors have posed hopes for sepsis therapy, as TLRs are key components in sepsis cascade.^[^ ^130^, ^131^, ^132^ ^]^

Several cationic lipids have been previously reported for their TLR4 modulating activity, including positively charged liposomes formed by cationic amphiphiles.^[^ ^133^ ^]^ For instance, diC14‐amidine liposomes have been shown to trigger the secretion of a cytokine pattern reminiscent of the TLR4‐dependent LPS secretion pattern by activating both MyD88/NF‐*κ*B/JNK and TRAM/TRIF pathways.^[^ ^134^ ^]^ Along similar lines, other cationic lipids have been found to promote cytokine production through NF‐*κ*B independent and TRIF‐dependent pathways, which require the presence of CD14.^[^ ^135^ ^]^ In addition, Piazza et al. demonstrated a specific binding of amino glycolipids and aromatic ammonium salts to CD14.^[^ ^136^, ^137^ ^]^ The targeting capability of these molecules toward CD14 inhibited the LPS‐stimulated TLR4‐dependent cytokine production in cells and animals.^[^ ^138^ ^]^ An interesting TLR modulatory nanodevice was also described by Rodriquez Lavado et al.^[^ ^139^ ^]^ AuNPs were coated with several cationic glycolipids, using the monosaccharide methyl *α*‐d‐glucopyranoside and the disaccharide *α*,*α*′‐trehalose as the sugar cores and assessed as TLR4 antagonists in vitro. The developed nanoplatforms showed strong inhibition of TLR4 activation on both human and murine LPS‐induced cells at cell tolerable concentrations. The TLR modulatory activity of the trehalose‐ and glucose‐derived glycoamphiphiles was attributed to the presence of acyl lipophilic chains at the hydrophobic domain as all compounds with ether bonds were inactive, while trehalose scaffold provided a well‐ordered facial amphiphilic character.^[^ ^139^ ^]^

Similarly, peptide‐AuNP hybrids were designed to serve as novel anti‐inflammatory agents by inhibiting TLR4 signaling (Figure 8E).^[^ ^140^ ^]^ Yang et al. screened a variety of peptide‐AuNP hybrids for successful TLR4 inhibition. Peptide‐AuNP hybrids with specific amino acid sequences inhibited the TLR4 signaling pathways and prevented from the excessive release of pro‐ and anti‐inflammatory cytokines. Their strong immunomodulatory activity was attributed to the hydrophobicity and aromatic ring structure of the amino acids. Yang et al. in a later study utilized the previously established peptide‐AuNP hybrids to regulate a broad spectrum of TLR signaling and responses, including TLR4 and TLR3.^[^ ^141^ ^]^ Interestingly, apart from controlling multiple TLR pathways, the improved nanoplatform suppressed the secretion of proinflammatory cytokines and chemokines. Further investigations revealed that the endosomal pH modulatory ability of peptide AuNPs was the mechanism by which the peptide enabled the downregulation of TLR signaling.

Another group used synthetic biomimetic NPs as inhibitors of inflammatory mediators induced by TLR4 binding to LPS.^[^ ^142^ ^]^ Inspired by the natural capacity of high density lipoprotein (HDL) to bind LPS^[^ ^143^, ^144^ ^]^ and the scavenging ability of NPs, Foit and Thaxton synthesized a library of HDL‐like NPs with AuNP core and HDL coating. One HDL‐like NP was particularly effective at decreasing TLR4 signaling triggered by the presence of LPS and Gram‐negative bacteria in human cells. The concentration of LPS was considerably reduced which in turn affected LPS‐mediated TLR4 activation.^[^ ^142^ ^]^

In a subsequent study, self‐assembling lipid modified non‐anticoagulant heparin NPs (NAHNP) were reported to serve as LPS inhibitors and TLR4 antagonists.^[^ ^145^ ^]^ Apart from its anticoagulant function, heparin can aid in the inhibition of inflammation‐involved proteins.^[^ ^146^ ^]^ Taking advantage of the antiinflammatory behavior of heparin, Babazada et al. developed lipid‐modified glycol‐split heparin NPs, which suppressed LPS‐induced inflammation through TLR4 and limited the production of cytokines.^[^ ^145^ ^]^

Cell‐free DNA (cfDNA) plays a pivotal role in the regulation of the Toll‐like receptor 9‐mediated proinflammatory cascade in severe sepsis.^[^ ^147^ ^]^ Thus neutralization of cfDNA may diminish the overwhelming immune response and benefit sepsis treatment. Inspired by this principle, Dawulieti et al. synthesized polyethylenimine (PEI)‐functionalized mesoporous silica NPs (MSNPs, 150 nm in size) which bound to proinflammatory nucleic acids and scavenged cfDNA.^[^ ^148^ ^]^ The scavenging activity of PEI‐MSNPs resulted in the inhibition of cfDNA‐triggered inflammation, the reduction of serum cytokines (TNF‐*α*, IL‐6, and MCP‐1) and elimination of organ damage. Histopathological and biochemical data of cecal ligation and puncture (CLP)‐induced severe sepsis mice revealed that injected PEI‐MSNPs accumulated and retained in the inflamed cecum, blocked the proinflammatory response and protected mice against multiple organ injury with negligible toxic effects in vivo.^[^ ^148^ ^]^ The NP‐based gene therapy described above, as well as the NP‐enable cell therapy described earlier,^[^ ^115^ ^]^ uncover the multitude of emerging opportunities nanomedicine can offer for the management of sepsis.

### 4.4 Immunomodulatory NPs in Sepsis

Apart from inhibiting TLRs, NPs have been also explored to serve sepsis immunotherapy, either by activating the host immune system to eliminate pathogens, or by suppressing cytokine‐producing immune cells.^[^ ^149^ ^]^ Considering the deregulation of the host immune system caused by the uncontrollable activation of immune cells during sepsis, targeting inflammatory cells to stimulate and control their apoptosis holds great therapeutic potential.^[^ ^150^ ^]^ On the other hand, total depletion of such cells using antibodies may exacerbate inflammation and jeopardize the innate and adaptive immune system. Thus, platforms that can specifically attack only the cytokine‐producing immune cells are urgently required and nanotechnology has already offered novel intracellular immunomodulatory systems.^[^ ^151^, ^152^ ^]^

In an interesting study by Zhang et al. doxorubicin (DOX)‐conjugated pH‐sensitive albumin NPs were synthesized to selectively target activated neutrophils and induce programmed cell death upon the release of DOX in vivo.^[^ ^151^ ^]^ As DOX is known to promote DNA damage‐associated cell death,^[^ ^153^ ^]^ authors hypothesized that it may also promote neutrophils apoptosis. In order to specifically attack inflammatory neutrophils, DOX was conjugated via hydrazine bonds with albumin NPs, which were internalized only by the activated neutrophils in circulation.^[^ ^151^ ^]^ Once NPs were taken up, the hydrazine bonds were degraded in the acidic environment of neutrophils allowing DOX leakage and in turn neutrophil apoptosis. In an LPS‐induced sepsis mouse model, 70% of mice survived in 72 h after being treated with DOX‐albumin NPs, while only 10% of those treated with free DOX survived. DOX‐albumin NPs treated mice had a significantly lower number of neutrophils and cytokines in their blood compared to PBS treated ones, indicating that the designed nanoplatform promoted the apoptosis of inflammatory neutrophils and suppressed systemic inflammation. Noteworthy, neutrophil numbers and cytokines of the NP‐treated mice recovered to their normal levels after 72 h, suggesting that no permanent damage occurred in the immune system and bone marrow function.

Tumor necrosis factor (TNF)‐related apoptosis‐inducing ligands (TRAIL), known to trigger inflammatory cells apoptosis, were encapsulated in an antimicrobial polypeptide‐crosslinked nanogel to suppress *K. pneumonia* infection and overexpressed MACs.^[^ ^152^ ^]^ The targeting capability of the proposed nanogels stemmed from their cationic fibril assemblies, which bound to the LPS compound of the bacterial wall through electrostatic interactions. Subsequently, nanogels were taken up by LPS‐activated MACs and TRAIL was released, promoting apoptosis. In a *K. pneumonia*‐induced sepsis mouse model, serum creatinine levels, blood bacterial load and the production of several cytokines (TNF‐*α* and IL‐6), kidney injury markers and LPS‐induced pulmonary polymorphonuclear leukocytes were significantly reduced compared with the free TRAIL, nanogel and saline controls. Furthermore, the survival rates of TRAIL‐nanogel treated mice were significantly improved, with almost 70% surviving in 12 days post‐treatment. Besides the impressive results of this study, mice were pretreated with the TRAIL‐nanogel, possibly affecting the therapeutic outcomes.

Exosomes, which are naturally secreted nanovesicles by cells, have been also proposed as immunomodulators as they carry miRNAs and proteins that regulate immune responses.^[^ ^154^ ^]^ In a study by Alexander et al. miR‐146a‐containing exosomes, released from bone marrow‐derived dendritic cells (BMDCs), were injected in LPS‐induced mice and downregulated inflammatory responses.^[^ ^155^ ^]^ Strikingly, a significant decline in the TNF‐*α* and IL‐6 serum levels of miR‐146a‐exosome‐treated mice was demonstrated compared with miR‐146a‐deficient exosome‐treated mice. Along similar lines, Wang et al. displayed that exosomal miR‐223 secreted by mesenchymal stem cells (MSCs) inhibited the uncontrollable release of inflammatory cytokines (TNF‐*α*, IL‐6, and IL‐1*β*) and protected CLP‐induced mice against cardiac dysfunction and death.^[^ ^156^ ^]^ The proposed mechanism behind the MSC‐induced immunomodulatory effects was thought to be the exosome‐mediated transfer of miR‐223 to MACs through exosomes internalization. Consequently, miR‐233‐containing exosomes suppressed inflammatory responses in MACs and cytokines storm was diminished.

These studies suggest that during sepsis, NP‐based immunomodulators can reprogram the immune system to block the uncontrollable secretion of inflammatory cytokines by specifically targeting inflammatory immune cells. Such strategies can open up new avenues toward the development of novel nano‐immunotherapeutics and contribute amply in sepsis management in ICU.

## 5 Sepsis Nanotheranostics

Recent design of nanoplatforms able to facilitate both diagnosis and therapy within a single nanoagent has prompted the rise of nanotheranostics.^[^ ^157^ ^]^ In sepsis, the simultaneous detection and killing of the causative agent by using a single nanoplatform can save valuable time and substantially contribute in the improvement of outcomes in the clinic.

As previously discussed, AuNPs unique optical and thermal properties have been extensively exploited for diagnostic purposes. Apart from their diagnostic utility, they have also proven to confer therapeutic potential for several diseases.^[^ ^158^, ^159^, ^160^, ^161^, ^162^, ^163^ ^]^ Near‐infrared adsorption of AuNPs allows efficient light‐to‐heat conversion, which contributes in deeper tissue penetration than other wavelengths of light, offering great advantages in photothermal (PT) therapy.^[^ ^164^ ^]^ In the context of sepsis, Zharov et al. developed an in vitro thermal‐based laser method for the simultaneous detection and killing of *S. aureus* using 10, 20, and 40 nm AuNPs conjugated with antiprotein A antibodies.^[^ ^165^ ^]^ Protein A is one of the major surface‐clustered proteins, which is linked to the peptidoglycan portion of the bacterial cell wall. Bacterial cells were incubated with antiprotein A‐AuNPs to allow NPs attachment to the bacterial wall. Due to their enhanced PT sensitivity, once irradiated and overheated, AuNPs nanoclusters caused irreparable bacterial damage.

Similar to AuNPs, carbon nanotubes (CNTs) have also been explored as promising nanotheranostic platforms in sepsis. The high binding affinity of CNTs to bacteria surfaces in addition to their strong absorbance in near‐infrared (NIR) and visible regions enable killing of bacteria by efficiently converting laser energy into strong thermal energy.^[^ ^26^ ^]^

**Figure 9:**
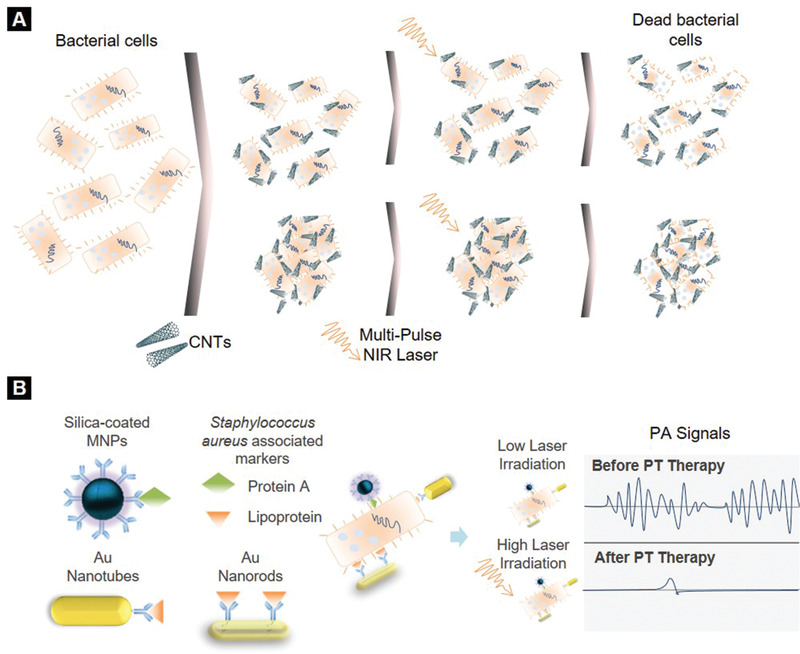
Nanotheranostic technologies for sepsis. A) Carbon nanotube (CNT)‐enabled photothermal detection and therapy of bacteria. CNT selectively bind to bacteria surface and form nanoclusters. Following multipulse NIR laser irradiation bacterial cells are killed. Laser energy is converted into thermal energy owing to CNTs powerful near‐infrared absorbance. Reproduced with permission.^[166]^Copyright 2007, Wiley Periodicals, LLC. B) PAFC/PTFL nanotheranostic platform for photoacoustic (PA) detection and photothermal (PT) treatment of circulating bacteria, taking advantage of optical, thermal and magnetic properties of gold (AuNPs) and magnetic nanoparticles (MNPs).

For example, CNTs were employed as contrast agents for the simultaneous detection and selective PT elimination of *E. coli* in vitro (**Figure** **9**A).^[^ ^166^ ^]^ Single‐walled (1.7 nm) and multiwalled (19.0 nm) CNTs were incubated with *E. coli* K12 strain for 0–2 h. Subsequently, *E. coli* samples with or without CNTs were irradiated with laser pulses and subjected to viability tests. At laser energy of 0.1 J cm^−2^ at 532 nm little or no damage was observed to the bacterium, while multipulse exposure of laser energy over 1 J cm^−2^ at 1064 nm and over 0.5 J cm^−2^ at 532 nm led to an 80% decrease in bacterial viability. Noteworthy, irreparable bacterial damage was observed at laser energy of 3 J cm^−2^ at 1064 nm and 2.5 J cm^−2^ at 532 nm. The high binding affinity of CNTs to bacteria, their ability to form self‐assembling nanoclusters on bacteria surfaces and their NIR strong absorbance, enabled the simultaneous detection and killing of *E. coli*. Nevertheless, according to the safety standards for medical laser, the maximum laser energy allowance is between 35 and 45 mJ cm^−2^ in NIR spectral range.^[^ ^166^ ^]^ These values are substantially lower than those described above, thus further optimization is needed to design systems that meet the required expectations.

In another interesting study, CNTs were used to enhance photoacoustic flow cytometry (PAFC) signals for in vivo real‐time monitoring of circulating *S. aureus* and *E. coli* in blood.^[^ ^167^ ^]^ Bacteria were labeled with CNTs upon a 30 h incubation. CNT‐labeled bacteria were then injected into the blood circulation of mice and monitored by PAFC (laser energy pulse 20–50 mJ cm^−2^). CNTs clustering amplified the thermal and acoustic power. Labeled‐bacteria were detected 1 min postinjection, while their elimination from the bloodstream started 3–5 min postinjection. Complete bacteria clearance was achieved 60 h after injection. This study provided with useful insight on CNTs effectiveness to generate ultrasensitive signals and simultaneously detect and eliminate single bacteria.

In view of the above, Galanzha et al. designed NP‐enabled PA/PT flow cytometry (PAFC/PTFC) platforms to facilitate in vivo magnetic enrichment, PA detection and PT elimination of circulating bacteria (Figure 9B).^[^ ^168^ ^]^ Silica‐coated MNPs, gold nanotubes (AuNTs) and gold nanorods (AuNRs) were functionalized with specific for *S. aureus* antibodies: protein A and lipoprotein. Bacteria were labeled with the antibody‐conjugated MNPs and AuNPs, were then injected into mice and monitored by PA/PT. Targeted PT therapy of bacteria at laser energy pulse of 0.8 mJ cm^−2^ at 850 nm led to a significant decrease in PA signal levels (laser energy 50 mJ cm^−2^), indicating bacterial growth inhibition. Due to the enhanced acoustic and thermal signals generated by the formed nanoclusters, circulating bacteria were monitored and killed with high sensitivity (0.5 CFU mL^−1^). This interesting study indicates that NPs‐PAFC/PTFC platforms could be the golden mean for the simultaneous detection and elimination of circulating pathogens associated with sepsis.^[^ ^169^ ^]^

More recently, Wang et al. developed a nanosystem to enrich single bacterial species in blood and simultaneously achieve complete extracorporeal disinfection.^[^ ^170^ ^]^ PEGylated iron oxide MNPs were coated with chlorin e6 molecules and aptamers (Fe_3_O_4_‐Ce6‐Apt, 17.2 nm) specific to *S. aureus*. *S. aureus*‐spiked blood samples were incubated with Fe_3_O_4_‐Ce6‐Apt and the number of bacterial cells was monitored in time. Fe_3_O_4_‐Ce6‐Apt revealed strong photostability under laser irradiation and enabled complete bacterial enrichment within 60 min with high sensitivity (10 CFU) and selectivity. Extracorporeal photodynamic blood disinfection took place by incubating *S. aureus*‐spiked blood samples with Fe_3_O_4_‐Ce6‐Apt for 1 h and irradiating the system with an NIR laser. Bacteria were successfully killed and then immediately cleared from blood by magnetic removal.

## 6 Future Research, Challenges, and Perspective

Nanomedicine research focuses on addressing the limitations of already existing technologies and creating novel diagnostic and therapeutic platforms. NPs are of immense scientific interest due to their unique properties which vary in respect to their bulk material. One example of the current research scenery is the research project “SmartDiagnos” at Denmark Technical University.^[^ ^171^ ^]^ Scientists developed a technology to identify sepsis bacterial infection within 3 h. Antibody immobilized magnetic nanobeads targeting specific bacterial genes were incubated with human plasma spiked with bacterial cells. Captured pathogens were identified by PCR with high sensitivity and low LOD (≈100 CFU mL^−1^). Strikingly, the integration of this approach into a lab‐on‐a‐chip biosensor is already envisioned to allow in‐depth blood analysis of pathogens.^[^ ^171^ ^]^

The recent release of a point‐of‐care diagnostic test for sepsis called “AbioSCOPE,” manufactured by Abionic SA, is another example of a clinically translated nanoplatform. This newly CE marked test is based on a patented nanofluidic technology and allows real‐time monitoring of patients at risk of sepsis by measuring pancreatic stone protein (PSP) levels in 5 min with high sensitivity (3.21 ng mL^−1^) and specificity. PSP is highly elevated during post‐traumatic sepsis and activates neutrophils.^[^ ^172^, ^173^, ^174^ ^]^ “AbioSCOPE” platform encompasses a fully automated fluorescent microscope and a mounting plate with a single‐use disposable capsule. Collected blood or serum is deposited into a capsule and automatically fills all nanofluidic biosensors by capillary action. PSP complexes are then formed and optically detected. At the moment, there are international ongoing clinical studies in over 200 ICU patients to evaluate the diagnostic performance of AbioSCOPE.

The bio‐barcode assay of Mirkin and Hill is another nanotechnological achievement, successfully clinically translated. Its evolution catalyzed the foundation of the Verigene System (Nanosphere Inc.), an automative reader that enables rapid and accurate detection of Gram‐positive bacteria and genes associated with bacterial infection.^[^ ^35^ ^]^ Besides diagnostics, nanotechnology has aided in the research and development of novel sepsis therapeutics with the design of cutting‐edge nanodevices,^[^ ^113^ ^]^ some of which have been successfully translated in the clinic (CytoSorb, CytoSorbents Corporation, Monmouth Jct., NJ).^[^ ^175^, ^176^, ^177^, ^178^, ^179^, ^180^ ^]^

Undoubtedly, nanomedicine can facilitate the development and application of diagnostic and therapeutic tools for sepsis. The replacement of current readout systems with portable devices which can offer automative diagnostic procedures and minimize user's intervention could likely be the key to accomplish clinical implementation. However, risks and challenges associated with the toxicity and long‐term safety of the emerging technologies are yet to be explored and thus time and persistent investment are required to allow clinical translation.

Furthermore, one should consider the possible implications that might arise when treating an inflammatory disorder with such complex immune responses. For instance, neutrophils release neutrophil extracellular traps (NETs) in response to infection by which they capture and kill pathogens extracellularly.^[^ ^181^ ^]^ These NETs, composed of chromatin fibers mixed with antimicrobial peptides and enzymes, are rapidly formed (<20 min) once the host is infected and have been shown to trap NPs, posing potentially a significant barrier in NP‐based drug delivery.^[^ ^182^, ^183^ ^]^ Some studies have also suggested that NPs themselves can trigger NET formation,^[^ ^183^, ^184^ ^]^ as neutrophils are involved in NPs clearance. When AuNPs of various surface chemistries, sizes and shapes were studied for their interaction with neutrophils, a time‐dependent‐NET formation was revealed with more NPs being trapped over an 1 h time course.^[^ ^183^ ^]^ Strikingly, NPs surface chemistry strongly impacted the amount of trapped NPs however, it could not prevent NET formation. Nanodiamonds and polystyrene NPs were also observed to trigger NETs formation in a size‐dependent manner, which inevitably led to their neutralization and entrapment.^[^ ^185^ ^]^ Designing thus NPs, which could either inhibit NET formation or could escape their entrapment is of the utmost importance. Interestingly, dextran and albumin coated SPIONs have been recently shown to prevent NET formation and vascular occlusions in vivo, suggesting that stabilization of NPs with biocompatible layers may lead to an inert behavior and phagocytosis‐induced clearance.^[^ ^186^ ^]^ Furthermore, as NETs are rapidly formed, treatment timing is crucial to successfully kill the infectious pathogens as early as possible and minimize the possibility of therapeutic failure due to NPs entrapment. Noteworthy, preliminary data from several NP‐based platforms has shown rapid elimination of pathogens even within 3–5 min of treatment,^[^ ^167^ ^]^ suggesting that NPs could act faster than NETs.

Finally, the translational gap between in vitro/ex vivo studies yielding positive results, animal‐based preclinical studies and clinical trials is another challenge that hampers the clinical translation of laboratory findings. One indicative example was the “ACCESS” trial which compared Eritoran, a TLR4 antagonist, to placebo in a double‐blinded randomized controlled trial.^[^ ^187^ ^]^ Strikingly, it was found that there was no reduction in the mortality of patients treated with the TLR4 antagonist compared to placebo at 28 days. Besides this trial did not include any NP‐based system, it still highlights the difficulty of scaling up and clinically implementing novel laboratory approaches for sepsis.

Indisputably, the clinical development of efficient, robust and biocompatible nanosystems either for diagnosis or treatment is a pathway full of challenges. Research should therefore focus on addressing the current limitations by designing and constantly evaluating new and innovative nanoplatforms.

## 7 Conclusion

Sepsis is a complex medical condition induced by dysregulated host response to an infection and without early treatment can lead to multiple organ failure and death. Despite technological advancements, sepsis incidents remain on the top 5 most frequent worldwide. During the last century, there is a tremendous effort to fight against sepsis and this has mobilized great interest and investment to support the development of novel diagnostic and therapeutic approaches. Nanotechnology can aid not only in antimicrobial treatment (Table 3) but also in the early diagnosis of sepsis. The use of NPs for sepsis diagnosis offers great advantages over conventional methods. Due to their unique properties and ease of functionalization to target specific pathogens, NPs provide higher diagnostic sensitivities than currently used routine methods and enable multiple‐species detection.

Despite recently obtained promising results, skepticism still prevails as to whether nano‐based technologies can be scaled up and clinically translated. The majority of laboratory techniques are far from clinical usage and implementation, while the biocompatibility and long‐term safety of diagnostic and therapeutic nanoplatforms have not been fully investigated. Additionally, the lack of clinical assessment hinders the development of robust and clinically applicable nanosystems. Hence, there is an imperative need for further optimization and clinical evaluation of the discovered technologies. The interdisciplinary merging of clinical, biological and physical sciences can vitally contribute in the translation of these encouraging nanotechnological breakthroughs into the clinic.

## Conflict of Interest

The authors declare no conflict of interest.
